# Learning epithelial elasticity via local tension remodeling

**DOI:** 10.1093/pnasnexus/pgag367

**Published:** 2026-10-25

**Authors:** Sadjad Arzash, Shiladitya Banerjee

**Affiliations:** School of Physics, Georgia Institute of Technology, Atlanta, GA 30332, USA; School of Physics, Georgia Institute of Technology, Atlanta, GA 30332, USA

**Keywords:** vertex model, physical learning, tension remodeling, elasticity

## Abstract

Biological materials such as epithelial tissues continuously remodel their internal structure in response to mechanical forces, but whether this adaptation amounts to a genuine form of physical learning has remained unclear. Here, we show that an active vertex model of epithelial sheets, equipped with a single local, mechanosensitive tension-remodeling rule, exhibits the three canonical hallmarks of learning matter. First, at the scale of a single junction, repeated contractile pulses produce habituation: a diminishing, history-dependent response that encodes a memory of prior stimulation. Second, at the tissue scale, a localized stimulus writes a long-range mechanical memory that biases the response to a later, distant stimulus. We derive a continuum theory showing that this memory is transmitted as a system-wide elastic bias and predict its dependence on tissue size and remodeling rate. Third, under cyclic bulk driving, the tissue can be trained to a target mechanical state, autonomously tuning its Poisson ratio to auxetic (negative) values through directed remodeling rather than designed geometry. We further derive an effective viscoelastic memory kernel that distinguishes this adaptive tissue from a passive vertex model. Together, these results identify a candidate mechanism for physical learning in epithelia, in which the ratio of contraction to extension remodeling rates acts as the central control parameter for programmable, history-dependent tissue-scale rheology.

Significance Statement Epithelial tissues continually remodel their cell–cell junctions in response to mechanical forces. Can this local adaptation store a mechanical memory and alter how a tissue responds to future forces? Using a model of epithelial sheets, we show that a single strain-dependent tension-remodeling rule produces three hallmarks of learning matter: the tissue habituates to repeated stimulation, stores a long-range memory of past stimuli, and can be trained by cyclic loading into targeted mechanical states, including an auxetic one that widens when pulled. The balance between remodeling during contraction and extension controls these responses. These results connect local junction remodeling to tissue-scale mechanical memory and suggest experiments to test and control history-dependent epithelial mechanics.

## Introduction

A growing class of physical systems can be trained. By storing memories of how physical systems are driven, they acquire functional properties without external design (1–8). Under repeated driving, these systems undergo irreversible microstructural changes so that their subsequent response encodes the training history (9–11). These ideas have been unified under a framework of “physical learning” or “learning matter” (12–18), in which fast degrees of freedom, such as node positions, are coupled to slow internal variables, such as bond stiffnesses or rest lengths, that record the loading history. These internal variables are updated by purely local rules, so the system adapts in a decentralized way, without global optimization or a prescribed task (19).

Such systems can exhibit a hierarchy of learning behaviors (2, 3). One of the simplest forms of learning is “habituation”: under repeated identical stimulation, the response progressively diminishes, a nonassociative form of learning. A second is “memory”: the system retains a durable imprint of past driving, as in the directed-aging protocol, where an elastic network is slowly driven while it relaxes, allowing local bond stiffnesses or rest lengths to evolve (6, 10). A sophisticated property is “trainability”: under a suitable protocol, the system is steered toward a target state, the canonical example being the cyclic training of a disordered network into an auxetic material with negative Poisson ratio (20). A central question is which physical systems implement which parts of this hierarchy.

We use the term “learning matter” in a sense that the system not only adapts to a stimulus but retains a history-dependent internal state that persistently modifies its future mechanical response. This distinguishes learning from mere adaptation, where the response to each stimulus is independent of prior loading, and from standard viscoelasticity, where the internal state is fully determined by the instantaneous configuration rather than its history.

Epithelial tissues are a compelling candidate for a biological realization of learning matter. As active, confluent sheets, their junctional tension, adhesion, and cortical forces are continuously remodeled by mechanochemical feedback in response to external and internal cues (21–29). Evidence from developing epithelia includes stress-dependent E-cadherin turnover and mechanically induced junction remodeling (25, 30). Such feedback provides a possible means of coupling fast mechanical relaxation to slower changes in junctional properties. Passive vertex models with fixed parameters reproduce baseline viscoelastic spectra and geometry-driven rigidity transitions (31–33) but cannot capture the history-dependent mechanical changes and remodeling observed in experiments (30, 34, 35). Adaptive models bridge this gap. Tuning cell shapes as additional degrees of freedom controls rigidity transitions, while local updates of edge tensions reproduce adaptive tissue flows during convergent extension (36, 37). Crucially, and unlike supervised protocols, this remodeling is driven intrinsically by local biophysical feedback (34), without an external cost function, which makes the epithelium a candidate for “autonomous” physical learning.

Here, we test whether an experimentally validated mechanosensitive tension-remodeling rule is by itself sufficient to make a model epithelial tissue behave as “learning matter,” a material that, through a single local rule, exhibits the characteristic signatures of physical learning—habituation to repeated stimuli, retention of long-range mechanical memory, and trainability of its global constitutive properties toward target states.

Building on our previously introduced framework of mechanical remodeling in epithelial junctions (21, 34, 35, 38, 39), we find that a single local tension-remodeling rule, with distinct rates for contraction ($k_{C}$) and extension ($k_{E}$), gives rise to three hallmarks of learning matter in succession. First, at the scale of a single junction, repeated contractile pulses produce “habituation”: the response diminishes with each pulse as the junction stores a permanent record of its loading history. Second, at the tissue scale, a short, localized contractile cue rewires the macroscopic mechanical properties, with the shear modulus increasing or decreasing depending on $k_{C}/k_{E}$, and writes a “long-range mechanical memory,” so that subsequent stimuli at distant sites elicit responses distinct from naïve tissues; we develop a continuum theory showing that this memory is transmitted as a system-wide elastic bias. Third, under small-amplitude cyclic bulk driving, the tissue becomes “trainable,” with its Poisson ratio tunable to auxetic (negative) values when $k_{C}>k_{E}$. We further derive an effective viscoelastic memory kernel that provides the analytical signature distinguishing this adaptive tissue from a passive vertex model. Conceptually, these findings identify a candidate local mechanism for physical learning in epithelial tissues, in which slow changes in junctional properties, directed by rapid, globally driven fields, yield targeted changes without centralized optimization.

## Methods

### Epithelial vertex model with mechanosensitive tension remodeling

We model epithelial tissue dynamics using a modified version of the standard 2D vertex model (32, 40–42). In this framework, each cell is represented as a polygon, and the degrees of freedom are the positions of the vertices shared between neighboring cells. The mechanical energy of the tissue is given by


(1) $$E=\sum_{\alpha =1}^{N}\left[K_{A}(A_{\alpha }-A_{0}{)}^{2}\right]+\sum_{\left\langle ij\right\rangle }\Lambda_{ij}\ell_{ij}+\Gamma_{a}\ell_{ij}^{2},$$

where the first summation runs over all cells, and the second over all edges between adjacent cells. Here, $A_{\alpha }$ and $A_{0}$ are the current area and the target area of cell *α*, and the coefficient $K_{A}$ controls the stiffness associated with area deformations. The second term describes the energy due to interfacial tension: each cell–cell junction *ij* of length $\ell_{ij}$ is associated with an active tension $\Lambda_{ij}(t)$, which evolves dynamically. The last term describes the energy due to actomyosin contractility, where $\Gamma_{a}$ is the contractile force per unit length of the edge (35, 38). The total tension on edge *ij* thus becomes $T_{ij}=\Lambda_{ij}+2\Gamma_{a}\ell_{ij}$. Position $r_{i}$ of vertex *i* evolves in time as: $\mu {\dot{r}}_{i}=-\partial E/\partial r_{i}$, where *μ* is the vertex friction coefficient.

The remodeling rule used here was derived from, and validated against, optogenetic experiments on epithelial junctions (34, 35). Junction contraction is reversible for weak or brief RhoA activation and permanent for strong or prolonged activation. Furthermore, ratcheting under pulsed activation was confirmed experimentally (34). While light sets when and where RhoA is activated, the remodeling is the cell’s own response, through E-cadherin clustering and endocytosis (34). Mechanosensitive junction remodeling also occurs without optogenetic input. For instance, E-cadherin turnover increases with stress in the developing *Drosophila* wing (25), and embryonic junctions remodel under direct optical manipulation (30). In this model (35, 38), the tension $\Lambda_{ij}$ along each cell–cell junction evolves according to


(2) $$\frac{d\Lambda_{ij}}{dt}=-k(\varepsilon_{ij})(\ell_{ij}-\ell_{ij}^{0})-\frac{1}{\tau_{\Lambda }}(\Lambda_{ij}-\Lambda_{0}),$$

where $\ell_{ij}$ is the instantaneous junction length, $\ell_{ij}^{0}$ is its rest length, and the strain is $\varepsilon_{ij}=(\ell_{ij}-\ell_{ij}^{0})/\ell_{ij}^{0}$. The second term describes tension relaxation to its equilibrium value $\Lambda_{0}$ over a timescale $\tau_{\Lambda }$. The tension remodeling rate $k(\varepsilon_{ij})$ depends on the strain as (see Fig. 1a)


(3) $$k(\varepsilon_{ij})=\left\{ \begin{array}{cc} k_{C}, & \varepsilon_{ij}<-\varepsilon_{c},\\ k_{E}, & \varepsilon_{ij}>\varepsilon_{c},\\ 0, & \text{otherwise}, \end{array}\right.$$

where $\varepsilon_{c}$ is a local threshold strain for junction remodeling. For positive $k_{C}$ and $k_{E}$, tension increases under contraction at rate $k_{C}$ and decreases under extension at rate $k_{E}$. The threshold $\varepsilon_{c}$ reflects the experimental observation that junctions remodel only above a critical strain amplitude, corresponding to sufficiently strong or sustained force (21, 35). Importantly, quantitative fits to the optogenetic experiments constrain the contraction-side remodeling rate to exceed the extension-side rate, $k_{C}>k_{E}$ (34, 35). We treat ${\tilde{k}}_{C}=k_{C}/k_{L}$ and ${\tilde{k}}_{E}=k_{E}/k_{L}$ as tunable parameters in order to map the full space of adaptive responses, but this experimentally determined asymmetry defines our primary regime of interest.

**Figure 1:**
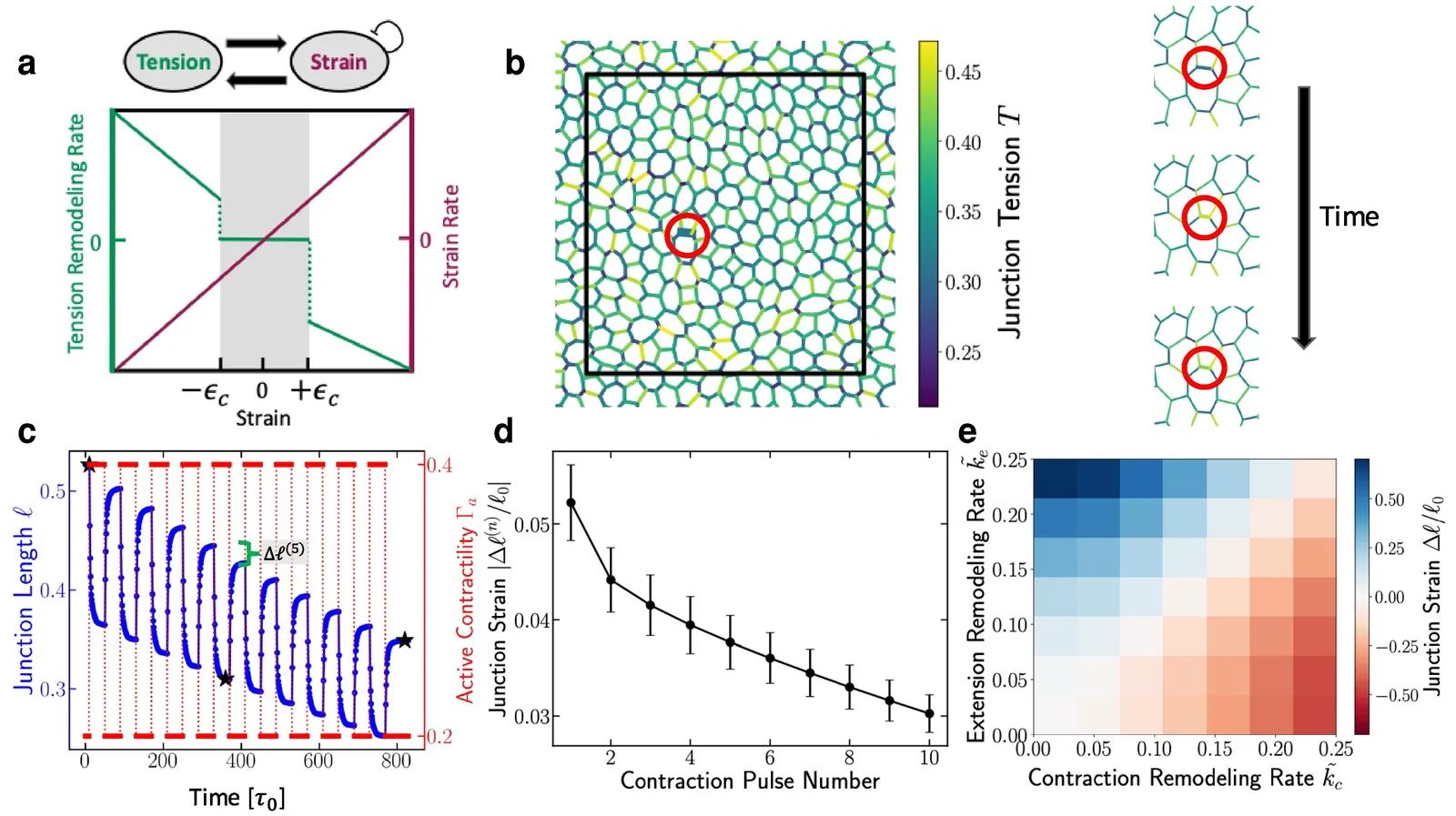
Adaptive dynamics of single junctions. a) Schematic of the tension-remodeling model. In the contracted regime ($\varepsilon <-\varepsilon_{c}$), edge tension increases at a rate $k_{C}$ while in the extension regime ($\varepsilon >\varepsilon_{c}$), the tension decreases at a rate $k_{E}$. The edge strain relaxes continuously at a rate $k_{L}$. b) A randomly selected edge in the tissue, with the colorbar indicating edge tensions. On the right: Three zoomed-in snapshots highlight the same edge at different times during application of a contractile active signal; the corresponding time points are marked with stars in (c). c) Time series of the selected edge length (left axis) under pulsatile contractile activations (right axis), illustrating how the edge progressively shortens with each activation. d) Net reduction in edge length as a function of the number of activations. Although repeated activations further reduce the edge length, the incremental reduction diminishes with each sequential activation, akin to habituation. e) Phase diagram of junctional strain as a function of ${\tilde{k}}_{C}$ and ${\tilde{k}}_{E}$ after the cessation of the contractile signal shown in (c).

In addition to tension remodeling, cellular junctions continuously relax strain through rest-length remodeling. The rest length $\ell_{ij}^{0}$ of each junction evolves toward its current length $\ell_{ij}$ at a rate $k_{L}$ (see Fig. 1a),


(4) $$\frac{d\ell_{ij}^{0}}{dt}=k_{L}(\ell_{ij}-\ell_{ij}^{0}),$$

representing the gradual turnover of strained actomyosin structures and their replacement by unstrained ones. While individual actomyosin structures turn over rapidly (e.g. with half-lives of 20 to 60 sec), the accumulation of mechanical memory in our model depends on a competition between timescales. The mechanosensitive remodeling rates ($k_{C},k_{E}$) must outpace this baseline tension relaxation rate ($\tau_{\Lambda }^{-1}$). If the relaxation rates are set to be faster than the remodeling rates, the tissue rapidly “forgets” the mechanical stimulus and no permanent reprogramming occurs.

An important consequence of Eq. 4 is that the tissue gradually forgets prior deformations over the characteristic timescale $k_{L}^{-1}$. Sustained contractions therefore remodel junctions only up to a finite limit, as strain is progressively relaxed, whereas pulsatile contractions interspersed with rest periods enable cumulative, irreversible shortening through ratcheting (21, 35). This interplay between tension remodeling and strain relaxation determines how persistent mechanical memory is written and erased in the tissue.

### Simulation methods

In the simulations, we nondimensionalize the equations of motion by setting $K_{A}=1$, $A_{0}=1$, and μ=0.1. Lengths are measured in units of $\sqrt{A_{0}}$, forces in units of $K_{A}A_{0}^{3/2}$, and time in units of the characteristic timescale $\tau_{0}=\mu /(K_{A}A_{0})$. With our parameter choices, one simulation time unit corresponds to a physical timescale of order ∼14s (38).

To generate the initial tissue geometry, we use the open-source CellGPU package (43). We place *N* random points as cell centers in a square simulation box of lateral size $L=\sqrt{N}$ with periodic boundaries, and construct the corresponding cellular network using a Voronoi tessellation. This yields a disordered confluent tissue composed of polygons with various numbers of neighbors. To obtain a mechanically relaxed reference state, we minimize the classical vertex-model energy


(5) $$E=\sum_{\alpha }K_{A}(A_{\alpha }-A_{0}{)}^{2}+K_{P}(P_{\alpha }-P_{0}{)}^{2},$$

with $K_{A}=K_{P}=1$, $A_{0}=1$, and $P_{0}=3.7$, placing the system in the solid-like regime. Here, $A_{\alpha }$ and $P_{\alpha }$ denote the instantaneous area and perimeter of cell *α*.

Each cell *α* is represented as an $n_{\alpha }$-sided polygon with ordered vertices $\{r_{m}{\}}_{m=1}^{n_{\alpha }}$ (with $r_{n_{\alpha }+1}\equiv r_{1}$). Its geometric measures are computed as


(6) $$A_{\alpha }=\frac{1}{2}\sum_{m=1}^{n_{\alpha }}(r_{m+1,x}+r_{m,x})(r_{m+1,y}-r_{m,y}),$$

(7) $$P_{\alpha }=\sum_{m=1}^{n_{\alpha }}\ell_{m,m+1},\;\ell_{m,m+1}=\|r_{m+1}-r_{m}\|.$$

We then relax the tissue using the FIRE algorithm (44) to obtain the minimum-energy configuration. During all simulations, T1 transitions are triggered when an edge length falls below a prescribed cutoff value of $\ell_{T_{1}}=0.05$.

The resulting force-balanced configuration serves as the reference tissue. We then initialize the edge tensions $\Lambda_{ij}$ in our adaptive vertex model to match the tensions computed from this equilibrium configuration. We then run the overdamped dynamics of Eq. 1 together with tension dynamics Eq. 2 and rest-length dynamics Eq. 4 until the system reaches a steady state with uniform active tensions, $\Lambda_{ij}=\Lambda_{0}$. This state serves as the initial tissue configuration for all subsequent simulations.

In the results presented below, we work in the limit of slow tension relaxation compared to tension remodeling, $\tau_{\Lambda }^{-1}\ll k\sqrt{A_{0}}/\Lambda_{0}$. The effect of faster relaxation timescales $\tau_{\Lambda }$ is discussed below. All supplementary movies are described in Section S13.

## Results

### Emergent memory kernel

We first establish that the tension-remodeling model (Eqs. 2–4) is generically a system with memory in the sense required of learning matter. It produces a history-dependent viscoelastic response, encoded in a memory kernel, that is identically absent in passive vertex models and vanishes when remodeling is switched off.

Consider an edge *ij* in the remodeling-active regime ($|\varepsilon_{ij}|>\varepsilon_{c}$), where the remodeling rate takes a constant value $\bar{k}$ ($\bar{k}=k_{C}$ for contraction, $k_{E}$ for extension). We linearize the tension dynamics (Eq. 2) and rest-length dynamics (Eq. 4) around a reference state, treating the edge-length perturbation $\delta \ell_{ij}(t)$ as a prescribed input driven by the fast vertex dynamics (see Section S1). Integrating out the slow variables $\delta \Lambda_{ij}$ and $\delta \ell_{ij}^{0}$, the change in total edge force ($f_{ij}$) takes the form


(8) $$\delta f_{ij}(t)=2\Gamma_{a}\delta \ell_{ij}(t)-\bar{k}\int_{-\infty }^{t}K(t-t^{\prime }){\dot{\delta \ell }}_{ij}(t^{\prime })dt^{\prime },$$

where the memory kernel is


(9) $$K(\tau )=\frac{e^{-\tau /\tau_{\Lambda }}-e^{-k_{L}\tau }}{k_{L}-1/\tau_{\Lambda }},\;\tau >0.$$

The first term in Eq. 8 is the instantaneous elastic response from the contractility $\Gamma_{a}$; the second is the history-dependent contribution from the slow internal variables. The kernel K(τ) has two characteristic timescales: $k_{L}^{-1}$ (rest-length relaxation) and $\tau_{\Lambda }$ (tension relaxation). It vanishes at τ=0 and reaches a maximum at an intermediate time before decaying. The kernel encodes three essential features of the model:

- Passive limit. When $\bar{k}=0$ (no remodeling), the memory term vanishes identically and the tissue responds as a purely elastic material.
- Permanent memory. In the limit $\tau_{\Lambda }\to \infty$ (slow tension relaxation), the kernel saturates: $K(\tau )\to (1-e^{-k_{L}\tau })/k_{L}$. The tissue then behaves as a standard linear solid with a delayed elastic response built up over the rest-length relaxation time $k_{L}^{-1}$. A step deformation then produces a permanent tension change $\delta \Lambda_{\infty }=-\tilde{k}\;\delta \ell_{0}$, where $\tilde{k}=\bar{k}/k_{L}$ is the dimensionless remodeling rate. This is the regime in which the tissue retains a persistent mechanical memory.
- Memory erasure. When $\tau_{\Lambda }^{-1}$ is large (fast tension relaxation), K(τ) decays rapidly and the memory term becomes negligible. The tissue “forgets” past deformations, recovering passive elastic behavior.

After a step strain, the tissue stress relaxation predicted by Eq. 8 is $\sigma (t)/\sigma_{0}=1-(\bar{k}/2\Gamma_{a})K(t)$, exhibiting a two-timescale relaxation that is absent in the static control. The kernel K(τ) could therefore be interpreted as the analytical signature of learning in this system: it distinguishes a tissue that has accumulated a history-dependent internal state from one that merely responds to its instantaneous configuration, and its structure directly predicts the slow stress relaxation and hysteresis observed in trained tissues (Section S9).

### Habituation and memory encoding in single junctions

We first elucidate the mechanism of mechanical memory encoding at the scale of a single cell–cell junction, which is crucial for the tissue’s emergent collective behavior. To this end, we examine the mechanical response of a single junction subjected to a pulsatile $\Gamma_{a}(t)$ (Fig. 1b and c), simulating pulsatile contractions that are widely observed during morphogenesis (45–49). In the tension-remodeling model, these periodic stimuli produce a clear ratcheting effect: each pulse shortens the junction and the postpulse length remains below its prepulse value, consistent with epithelial junction experiments (34). Mechanistically, when the instantaneous strain crosses the negative threshold in (3), the update rule in (2) increases the junctional tension during the contracted phase. After the external contractility is removed, the junction relaxes toward a longer length, but the elevated tension (the stored memory) prevents full recovery, yielding a net shortening (Fig. 1c).

Successive pulses further increase tension and accumulate shortening, while the incremental shrinkage per pulse decreases over time (Fig. 1d). This diminishing response under repetitive stimuli is a signature of “habituation,” a simple form of nonassociative learning (50). The diminishing per-pulse response arises from rest-length adaptation: after each pulse, $\ell_{ij}^{0}$ relaxes toward the contracted junction length at rate $k_{L}$, progressively reducing the strain mismatch $\varepsilon_{ij}$ that drives tension remodeling in subsequent pulses. The same process that writes mechanical memory thereby limits further memory accumulation, producing the self-limiting response characteristic of habituation.

The direction and magnitude of the permanent length change ($\Delta \ell /\ell_{0}$) are controlled by the two dimensionless tension remodeling rates, ${\tilde{k}}_{C}=k_{C}/k_{L}$ (contraction remodeling rate) and ${\tilde{k}}_{E}=k_{E}/k_{L}$ (extension remodeling rate). This ratio acts as the fundamental control parameter for local mechanical adaptation (Fig. 1e). When the contraction remodeling rate dominates (${\tilde{k}}_{C}>{\tilde{k}}_{E}$), pulses drive a net shortening. Whereas, when the extension remodeling rate dominates (${\tilde{k}}_{E}>{\tilde{k}}_{C}$), the same contractile pulses can surprisingly produce a net lengthening of the junction. In Section S2, we show how an edge successively lengthens under a pulsatile contractile signal in the regime ${\tilde{k}}_{E}>{\tilde{k}}_{C}$. This behavior is summarized in the phase diagram of the junctional strain $\Delta \ell /\ell_{0}$ (Fig. 1e), which delineates the shortening and lengthening regimes in the $\left({\tilde{k}}_{C},{\tilde{k}}_{E}\right)$ plane. The ratio ${\tilde{k}}_{C}/{\tilde{k}}_{E}$ determines the direction of the final structural change. Moreover, the net edge length change depends sensitively on the pulse period: for short periods the length change is negligible, whereas increasing the period produces larger changes that eventually saturate at a plateau value for this signal (Section S3). Based on experimental data (35, 51), we expect epithelial tissues typically operate in the regime where the contraction-induced remodeling rate is higher (${\tilde{k}}_{C}>{\tilde{k}}_{E}$), favoring net shortening and stiffening. The single-junction habituation establishes a key hallmark of learning matter and reveals the mechanism underlying it: whenever a slow memory variable sets the reference for the stimulus driving its own evolution, habituation is structurally encoded in the update rule.

### Writing mechanical memory: Local cues reprogram tissue-scale elasticity

We next ask whether the junctional memory established by habituation accumulates into a durable, tissue-scale imprint. Specifically, we ask whether localized, pulsatile contractility can permanently reprogram tissue-scale material properties. To this end, we apply contractile pulses to a circular region of radius *R* (Fig. 2a) and track how local junctional remodeling alters the tissue’s global mechanical response (Movie S1). Localized contraction pulses generate nonlocal changes in tissue properties via memory propagation through the junctional network (Fig. 2a). The permanent, history-dependent changes accumulated across the entire network result in a stable, reprogrammed mechanical state, which we quantify by measuring the linear shear modulus (*G*) of the fully relaxed tissue.

**Figure 2:**
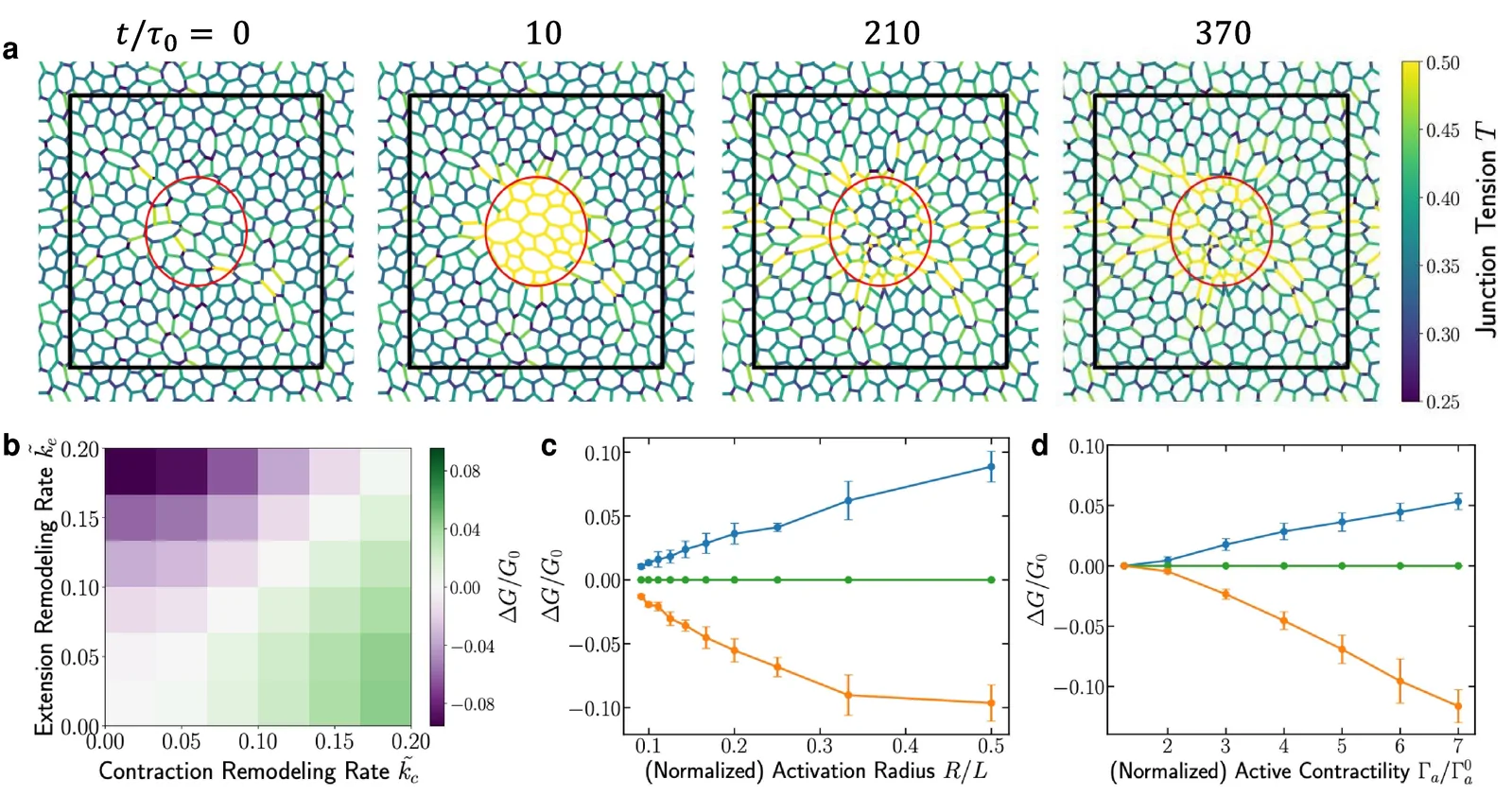
Local junction remodeling reprograms global mechanical properties. a) Simulation snapshots of a circular activation region of radius *R* at the tissue center. The adjacent scale indicates junction tensions. The first snapshot shows the initial configuration with no contractile signal. The second snapshot shows the response during activation, where edges within the region develop high tension. The remaining snapshots illustrate the relaxation after the signal is removed, revealing that high-tension edges persist and spread into surrounding cells. These dynamics demonstrate that a locally applied contractile signal propagates through the tissue via feedback between tension and strain fields. b) Phase diagram of the change in tissue shear modulus after five consecutive contractile activations applied to a central region of size R/L=1/6. The applied signal matches that of Figure 1, with $\Gamma_{a}^{0}=0.2$ (low contractility), $\Gamma_{a}=1.2$ (high contractility), and pulse duration 40. c) Change in shear modulus at $\Gamma_{a}/\Gamma_{a}^{0}=4$ as a function of region size R/L. The increasing curve corresponds to ${\tilde{k}}_{C}=0.2$, ${\tilde{k}}_{E}=0$; the zero-change curve to ${\tilde{k}}_{C}={\tilde{k}}_{E}=0$; and the decreasing curve to ${\tilde{k}}_{C}=0$, ${\tilde{k}}_{E}=0.2$. d) Change in shear modulus at R/L=1/6 as a function of the contractility ratio $\Gamma_{a}/\Gamma_{a}^{0}$, for the same three remodeling conditions as in (c).

To compute the shear modulus *G* of the dynamic tension-remodeling model, we first arrest all dynamical updates of the edge tensions $\Lambda_{ij}$. For a given tissue configuration, the system is then relaxed to a mechanically stable state by minimizing the tension-remodeling vertex-model energy in (1). The minimization is performed using the FIRE algorithm, following the procedure used above for preparing relaxed initial states.

Once the mechanically stable configuration is obtained, we compute the shear modulus by evaluating the second total derivative of the energy with respect to an infinitesimal simple shear strain *γ*. For each configuration, we apply an affine simple shear of the simulation box with amplitudes γ=−Δγ,0,+Δγ, updating all vertex positions accordingly. After each shear step, the vertex positions are relaxed using the FIRE algorithm while keeping $\Lambda_{ij}$ fixed, and we record the corresponding energies E(−Δγ), E(0), and E(+Δγ). The shear modulus is then obtained from a centered finite-difference approximation to the second derivative,


(10) $$G=\frac{1}{L^{2}}\frac{E(+\Delta \gamma )-2E(0)+E(-\Delta \gamma )}{\Delta \gamma^{2}}.$$

We have verified that the resulting *G* is insensitive to the choice of a small Δγ, confirming that the calculation is performed in the linear elastic regime. We note that this numerical procedure is consistent with the standard definition of the linear shear modulus obtained from the Hessian of the system (52, 53). Because the tension degrees of freedom are held fixed during these minimizations, this procedure isolates the instantaneous elastic response of a given trained state of the tissue.

We report the change in shear modulus, $\Delta G=G_{\mathrm{final}}-G_{\mathrm{initial}}$, after a fixed number of active contractile pulses. The direction of the global change (ΔG) is determined by the imbalance of the local remodeling rates, ${\tilde{k}}_{C}$ and ${\tilde{k}}_{E}$, as shown in the phase diagram in Fig. 2b. This ratio serves as the global control knob for programming the tissue’s stiffness. Specifically, for ${\tilde{k}}_{C}>{\tilde{k}}_{E}$, contraction-side remodeling dominates, and the tissue exhibits stiffening (ΔG>0). Conversely, for ${\tilde{k}}_{E}>{\tilde{k}}_{C}$, extension-side remodeling dominates, leading to tissue softening (ΔG<0). In the passive limit where remodeling is absent (${\tilde{k}}_{C}={\tilde{k}}_{E}=0$), ΔG=0, confirming that the observed changes arise solely from tension remodeling. Since fits to optogenetic experiments place epithelial junctions in the regime ${\tilde{k}}_{C}>{\tilde{k}}_{E}$ (34, 35), we predict that localized active pulses stiffen the tissue. This offers a concrete, testable hypothesis using optogenetic tools combined with rheological experiments.

The magnitude of ΔG is highly tunable. Under a fixed number of pulses, |ΔG| increases approximately linearly with the size of the activated region for small R/L (Fig. 2c). At fixed *R*, |ΔG| also grows with pulse amplitude $\Gamma_{a}$ (Fig. 2d). Thus, localized contractions provide a control knob to program global mechanical properties, stiffening or softening, by tuning the balance of remodeling rates and the spatial extent and strength of the driven region. Additional analysis, performed at fixed relative activation size $R/\sqrt{N}=1/6$, shows that the normalized stiffness change exhibits finite-size effects for small tissues but approaches a plateau at larger *N* (Section S4).

### Long-range mechanical memory drives cooperative response

Having established that local tension remodeling writes a durable mechanical memory in the global shear modulus, we ask how far this memory reaches: whether the imprint of a localized stimulus extends across the tissue to bias responses at distant sites, a signature of long-range memory.

To address this, we designed a two-stage simulation. We first applied a localized contraction pulse to a “trained” region (region 1) of the tissue and allowed the system to mechanically relax (Fig. 3a). We then applied a second, identical pulse to a distant, “test” region (region 2), separated by distance *D* (Fig. 3b, Movie S2). We quantify the memory effect by comparing the average active tension generated in region 2 under two conditions: (i) when region 1 was previously activated (green curve, Fig. 3c) vs. (ii) when region 1 was naive (black curve, Fig. 3c). The results show that the average tension in region 2 rises more rapidly and reaches a higher final value when the tissue has previously experienced an active contraction elsewhere (Fig. 3c).

**Figure 3:**
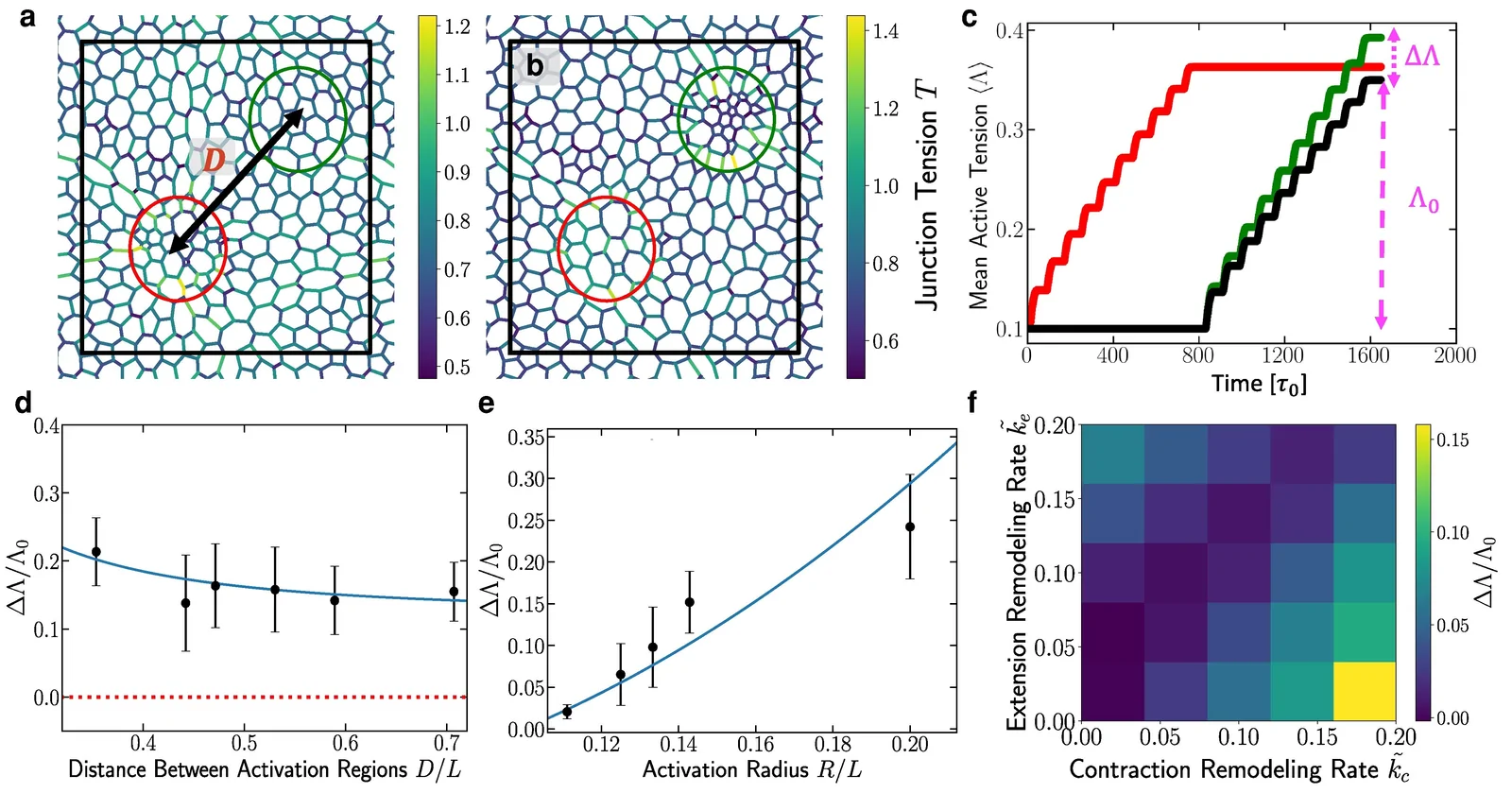
Long-range cooperative response from local contractile stimulus. a) Tissue configuration after pulsatile activation of the first circular region, located at the lower left, followed by removal of the signal. Unless otherwise stated, the activated regions have radius R/L=1/6, and the applied signal matches that of Fig. 1, with baseline contractility $\Gamma_{a}^{0}=0.5$, $\Gamma_{a}=2$, and pulse period 40. b) Tissue configuration after subsequently applying the same pulsatile signal to the second circular region, located at the upper right, and removing the signal. The prior activation of the first region leaves a mechanical memory in the tissue that modifies the response of the second region. Mean active tension in the first region during the earlier pulse sequence and in the second region during the later pulse sequence. For the second region, the upper trajectory shows the response after prior activation of the first region, and the lower trajectory shows the control response without that prior activation. The difference between the final mean active tensions in the second region with and without prior activation of the first region defines the “cooperative enhancement”. Its normalized value is plotted against the center-to-center separation of the activated regions. The solid curve is a fit to Eq. 13, with R=L/6, showing a finite large-distance plateau with a weak distance-dependent correction. e) Cooperative enhancement plateau as a function of activation radius *R* at fixed separation D/L=0.7. The solid curve is a fit to $a(R/L{)}^{2}+b$. The $(R/L{)}^{2}$ dependence is the continuum prediction, Eq. 12, and the offset b=−0.12 is not captured by the continuum theory. f) Cooperative enhancement phase diagram as a function of ${\tilde{k}}_{C}$ and ${\tilde{k}}_{E}$.

We define the cooperative enhancement (ΔΛ) as the increase in the final average active tension (*Λ*) in region 2 caused by the prior activation of region 1 (Fig. 3c). The enhancement is cooperative because a prior stimulus amplifies the tissue’s response to a later one, and “long-range” because, as we show below, it persists even when the two regions are far apart.

To determine the spatial extent of this memory, we systematically varied the center-to-center distance *D* between the two activated regions (Fig. 3a). As shown in Fig. 3d, the memory effect (ΔΛ) decays slowly with increasing distance *D* but saturates to a finite, nonzero value $\Delta \Lambda_{\infty }$, which increases with the size of the activation region (Fig. 3e). This suggests that the interaction is not purely local, such as through direct cell–cell contact but is instead mediated through long-range mechanical coupling in the whole tissue. The permanent tension and rest-length changes stored locally in region 1 globally bias the elastic response of the tissue, making the distant region 2 more susceptible to remodeling upon activation.

The strength of this cooperative effect further depends on the rates of contraction (${\tilde{k}}_{C}$) and extension (${\tilde{k}}_{E}$) induced tension remodeling (Fig. 3f). This dependence shows that the ${\tilde{k}}_{C}/{\tilde{k}}_{E}$ ratio not only dictates the direction of adaptation but also amplifies the magnitude of the cooperative enhancement. Specifically, for the experimentally relevant regime where ${\tilde{k}}_{C}>{\tilde{k}}_{E}$, increasing ${\tilde{k}}_{C}$ at a fixed ${\tilde{k}}_{E}$ increases the cooperative enhancement (ΔΛ) (Fig. 3f). This amplification occurs because the initial stimulus (region 1 activation) creates a large number of high-tension, memory-encoding junctions. A higher ${\tilde{k}}_{C}$ ensures that the local tension accumulated in these junctions is greater and more stable, resulting in a globally stiffer initial state for the tissue. When region 2 is then activated, the enhanced stiffness transmits a stronger baseline stress through the network, pushing the local strain in region 2 more quickly past the remodeling threshold and accelerating its own tension accumulation.

### Origin of long-range cooperative response

To explain the physical origin of cooperative tension enhancement (Fig. 3c–f), we derived a coarse-grained continuum description of the vertex model (see SI, Section S5). In this framework, we can treat the activated region 1 as a mechanical inclusion in a finite elastic sheet (54). After region 1 is activated and the tissue has fully relaxed, the permanently elevated tensions $\Lambda_{ij}=\Lambda_{0}+\delta \Lambda_{\mathrm{src}}$ inside region 1 act as an isotropic eigenstress source, where $\delta \Lambda_{\mathrm{src}}>0$ is the net tension accumulated through junctional ratcheting. The isotropy follows from the random orientations of the many edges inside the activated region, which average to a pure pressure perturbation $\delta \sigma_{mn}^{\mathrm{src}}=p_{0}\delta_{mn}\Theta_{R}(r)$, where $p_{0}=n_{e}\delta \Lambda_{\mathrm{src}}\ell_{0}/2$ is the eigenstress amplitude, $n_{e}$ is the edge number density, and $\Theta_{R}(r)$ is the indicator function of region 1.

Treating the tissue as a 2D isotropic elastic continuum with bulk modulus *B* and shear modulus *G* (derived from the vertex-model energy; see Section S5), and enforcing area conservation in the L×L periodic box, we solve the Navier equation sourced by $-p_{0}\nabla \Theta_{R}$. Because the source is purely longitudinal (the gradient of a scalar), the resulting displacement field is irrotational, and the dilation field takes an exact, closed form (Section S5):


(11) $$\nabla \cdot u(r)=\left\{ \begin{array}{cc} -\frac{\;p_{0}(1-\phi )}{B+G}, & |r|<R,\\ +\frac{\;p_{0}\phi }{B+G}, & |r|>R, \end{array}\right.$$

where u is the displacement field, $\phi =\pi R^{2}/L^{2}$ is the activated area fraction and B+G is the 2D longitudinal modulus. The compression inside region 1 is compensated by a spatially uniform dilation of the surrounding tissue, independent of position. This is the 2D Eshelby result for a circular pressure inclusion, where the strain outside a circular isotropic eigenstress region is exactly uniform.

When region 2 is subsequently activated at distance *D*, every edge inside it starts from a predilated background strain $\varepsilon_{\mathrm{bg}}=p_{0}\phi /[2(B+G)]$ that is independent of *D*. This uniform prestrain biases the remodeling dynamics during region 2’s activation, producing a cooperative enhancement


(12) $$\Delta \Lambda_{\infty }=\frac{\eta p_{0}\phi }{2(B+G)}=\frac{\eta n_{e}\delta \Lambda_{\mathrm{src}}\ell_{0}}{4(B+G)}\cdot \frac{\pi R^{2}}{L^{2}},$$

where $\eta =\partial \Lambda_{\mathrm{final}}/\partial \varepsilon_{\mathrm{bg}}$ is a mechanosensitive susceptibility that quantifies how efficiently a uniform background strain is converted into additional tension during activation. The plateau (see Fig. 3d) is controlled by three groups of parameters: (i) how much tension memory region 1 deposits ($\delta \Lambda_{\mathrm{src}}$, set by $k_{C}$, $\Gamma_{a}$, and the number of pulses); (ii) the elastic transmissivity of the tissue (B+G, set by $K_{A}$, $\Lambda_{0}$, and $\Gamma_{a}$); and (iii) the activated area fraction $\phi =\pi R^{2}/L^{2}$. Vertex friction *μ* does not appear, since the strain field is measured at mechanical equilibrium. When tension relaxation is fast ($\tau_{\Lambda }^{-1}$ large), $\delta \Lambda_{\mathrm{src}}$ itself decays before region 2 is activated and the plateau vanishes.

In the discrete vertex model, the eigenstress has a small deviatoric (anisotropic) component from network disorder, which generates a distance-dependent correction $∼(R/D{)}^{2}$ (see Section S5). The full cooperative enhancement is therefore


(13) $$\Delta \Lambda (D)=A{\left(\frac{R}{D}\right)}^{2}+\Delta \Lambda_{\infty }.$$

The weak distance-dependence observed in simulations (Fig. 3d) reflects the dominance of the isotropic component. The many randomly oriented edges in region 1 suppress the deviatoric eigenstress by $∼1/\sqrt{N_{\mathrm{edges}}}$, leaving the distance-independent dilation as the leading contribution. The plateau scales as $\phi =\pi R^{2}/L^{2}$ (see Fig. 3e) and vanishes in the thermodynamic limit (L→∞ at fixed *R*), while the $A(R/D{)}^{2}$ term persists. Thus, cooperative enhancement in an infinite tissue is algebraically long-ranged, while in finite tissues it acquires an additional system-size-dependent plateau from the global area-conservation constraint.

### Trainability: Programming auxeticity via cyclic deformations

Having demonstrated habituation and long-range memory, we now ask whether the tissue displays trainability, the ability to be steered toward a target mechanical state. A particularly relevant property to train is the Poisson’s ratio (*ν*), which quantifies the transverse response of a material under uniaxial strain. Materials with a negative Poisson’s ratio (auxetic materials) expand laterally when stretched and contract laterally when compressed (20, 55–57). Recent studies have shown that periodic mechanical training, cyclically deforming disordered networks, can tune their internal structure to produce auxetic behavior through local remodeling (20). Here, we show that epithelial-like adaptive networks can achieve the same effect autonomously, through mechanosensitive tension remodeling at cell junctions.

To test this programmability, we subject the tissue to externally imposed oscillatory bulk deformations of amplitude $\varepsilon_{B}$ (Fig. 4a, Movie S3). During each cycle of isotropic compression and expansion, all cell edges experience local strain, while the adaptive variables, edge tensions $\Lambda_{ij}$ and rest lengths $\ell_{ij}^{0}$, evolve according to (2) and (4). These deformations act as a mechanical training protocol that gradually tunes the microscopic edge properties and, consequently, the emergent elastic response of the tissue. After applying a fixed number of bulk-training cycles, we relax the system to mechanical equilibrium and measure its effective Poisson’s ratio. To compute *ν*, we impose a small uniaxial strain $\varepsilon_{y}$ along the *y*-axis, allow the tissue to relax, and measure the induced transverse strain $\varepsilon_{x}$ along the *x*-axis. The Poisson’s ratio is then calculated as $\nu =-\varepsilon_{x}/\varepsilon_{y}$ (Fig. 4a).

**Figure 4:**
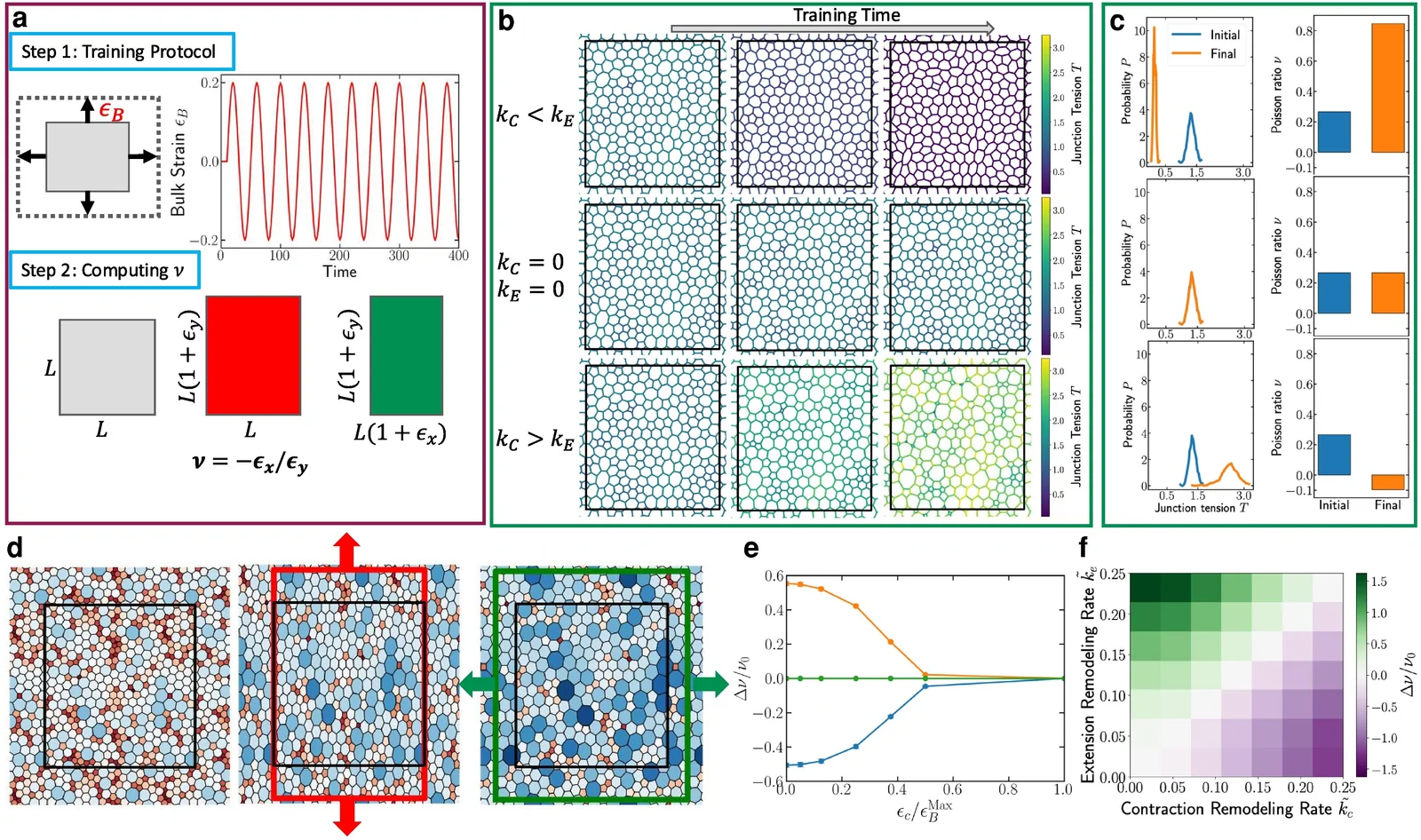
Training auxetic states under periodic bulk deformations. a) Training tissue’s Poisson ratio under sinusoidal bulk deformations. Upper left panel: schematic of a bulk deformation with magnitude $\epsilon_{B}$. Upper right: sinusoidal bulk-deformation signal applied over time. Lower panel: Schematic of the method used to compute the Poisson ratio. b) Tissue snapshots under bulk deformation. Top row: evolution from the initial state to mid-training and the final configuration for ${\tilde{k}}_{C}<{\tilde{k}}_{E}$. Middle row: case with ${\tilde{k}}_{C}={\tilde{k}}_{E}=0$. Bottom row: case with ${\tilde{k}}_{C}>{\tilde{k}}_{E}$. c) Initial and final edge-tension distributions for the three conditions shown in (b), with the corresponding initial and final Poisson ratios in the adjacent bar charts. Extension-dominated remodeling shifts the distribution toward lower tensions, the distributions overlap without remodeling, and contraction-dominated remodeling broadens the distribution toward higher tensions. d) Auxetic response of a tissue under uniaxial stretch. Cell shading represents the area deviation $\Delta A_{\alpha }=A_{\alpha }-A_{0}$, positive values indicate areas larger than the target area and negative values indicate smaller areas. Left: undeformed reference state. Middle: tissue under an imposed deformation along the *y*-direction. Right: relaxed configuration showing lateral expansion of the *x*-boundary, consistent with a negative Poisson ratio. For visual clarity, this example uses a relatively large applied deformation; all reported values of the linear Poisson ratio *ν* are computed using a small strain of 1%. e) Change in Poisson ratio as a function of the ratio of the critical edge-level strain to the maximum applied bulk strain, $\epsilon_{c}/\epsilon_{B}^{\mathrm{Max}}$. Orange: ${\tilde{k}}_{C}=0$, ${\tilde{k}}_{E}=0.2$; green: ${\tilde{k}}_{C}={\tilde{k}}_{E}=0$; blue: ${\tilde{k}}_{C}=0.2$, ${\tilde{k}}_{E}=0$. f) Phase diagram for the case $\epsilon_{c}=0.01$ and $\epsilon_{B}^{\mathrm{Max}}=0.2$.

Figure 4b shows representative tissue configurations and tension fields for three regimes: (i) $k_{C}<k_{E}$, (ii) $k_{C}=k_{E}=0$, and (iii) $k_{C}>k_{E}$. When $k_{C}<k_{E}$, edge tensions decrease over repeated bulk cycles, leading to more uniform cell areas (Section S6) and an increase in *ν*. In the passive control model without remodeling ($k_{C}=k_{E}=0$), both tensions and *ν* remain unchanged. By contrast, when $k_{C}>k_{E}$, edge tensions grow monotonically during training, generating strong structural and tension heterogeneity with coexisting large and small cells (Fig. 4b and c; Section S6). This highly tensed, heterogeneous architecture drives *ν* downward, ultimately yielding negative values characteristic of an auxetic response (Fig. 4c and d).

The ${\tilde{k}}_{C}/{\tilde{k}}_{E}$ ratio acts as the primary remodeling asymmetry, controlling the direction and magnitude of the programmed change in *ν*, as shown in the phase diagram (Fig. 4f). Additionally, the training is gated by the ratio of the applied strain to the local remodeling threshold ($\varepsilon_{B}/\varepsilon_{c}$). When the applied deformation $\varepsilon_{B}$ is too small, the tissue is inert; as the ratio $\varepsilon_{B}/\varepsilon_{c}$ increases, adaptive remodeling becomes stronger, and the magnitude of the programmed change in *ν* grows rapidly (Fig. 4e).

These results demonstrate that epithelia-like adaptive networks can be trained through repeated bulk deformations to acquire programmable elastic responses, including negative Poisson’s ratios, without any designed geometry or plastic flow, purely through dynamic mechanosensitive feedback. In the regime $k_{C}>k_{E}$, our theory suggests that under oscillatory external deformations, tissues would continuously stiffen and decrease their Poisson ratio. This presents an intriguing experimentally testable prediction for epithelial monolayers. We verified that the trained change in *ν* is robust to the T1 cutoff length and to different post-T1 assignment rules over the range studied (Section S7). We further verified that the trained auxetic response is robust to stochastic junctional tension fluctuations over the range studied (Section S12).

The microscopic origin of this trained auxeticity is a tension-driven rigidity switch, which can be captured analytically with a minimal mean-field model. Consider a regular hexagonal cell governed by the vertex-model energy:


(14) $$E_{\mathrm{hex}}=K_{A}(A-A_{0}{)}^{2}+\Lambda P.$$

Under a small uniaxial strain $\varepsilon_{yy}$, the cell deforms transversely by $\varepsilon_{xx}$ to minimize this energy. The linear Poisson ratio, $\nu =-\varepsilon_{xx}/\varepsilon_{yy}$, is fundamentally controlled by the dimensionless junctional tension $\tilde{\Lambda }=\Lambda /(K_{A}A_{0}^{3/2})$.

As shown in Fig. S8, increasing the dimensionless junctional tension $\tilde{\Lambda }$ systematically lowers *ν* and eventually drives it negative (Section S8). In the naïve (untrained) tissue, $\tilde{\Lambda }$ is small, and the stiff area elasticity term dictates the mechanical response. To maintain the target cell area $A_{0}$ under vertical stretch, the cell must contract laterally, yielding a standard positive Poisson ratio (ν>0). However, when the cyclic training protocol operates in the contraction-dominated regime ($k_{C}>k_{E}$), the internal remodeling rule progressively increases *Λ*. Once $\tilde{\Lambda }$ exceeds a critical threshold ${\tilde{\Lambda }}_{c}$, the perimeter’s energetic penalty dominates. To minimize this massive tension under vertical stretch, the vertices surrounding the stretched cells are forced to move outward laterally against the softer area constraint, causing an expansion that yields a negative Poisson ratio (ν<0). Thus, auxeticity is a fundamental, physically accessible target state, autonomously reached through local tension remodeling without any external cost function or designed microstructure, which is a defining characteristic of trainability in learning matter.

Importantly, a trained tissue is not equivalent to a static high-tension vertex model: even when the static control is matched to the trained tissue’s edge-tension distribution, the trained tissue shows different spatial organization of tensions and distinct mechanical responses, including slow stress relaxation and hysteresis (Section S9). The auxetic response itself arises through an organized, heterogeneous nonaffine relaxation of the trained network rather than a uniform affine expansion, concentrated at interfaces between small and large cells (Section S10).

## Discussion

Our results show that a minimal, mechanosensitive tension-remodeling rule is sufficient to make an epithelial tissue model behave as learning matter, exhibiting in succession the three canonical hallmarks of physical learning: habituation to repeated stimuli, long-range mechanical memory, and trainability to a target mechanical state. The same local rule that quantitatively captures optogenetic junction experiments (34, 35) gives rise to all three hallmarks. The rule is experimentally established at the junction level. The hallmarks are predictions to be tested in living epithelia.

The model identifies two primary control levers for programming the material. First, the imbalance between contraction- and extension-side remodeling sets the direction and magnitude of mechanical adaptation. When contraction-driven remodeling rate is higher ($k_{C}>k_{E}$), localized pulses stiffen the material and cyclic bulk driving lowers *ν*, switching the material to auxetic behavior. When extension-induced remodeling rate is higher ($k_{E}>k_{C}$), the opposite trends occur. Second, the amplitude of the applied strain relative to a local strain threshold for junctions ($\varepsilon_{B}/\varepsilon_{c}$) gates the adaptive response: for $\varepsilon_{B}<\varepsilon_{c}$, the epithelial sheet is inert; above threshold, per-cycle updates add coherently and the trained response grows.

In living systems, the rapid turnover of actin and myosin means that persistent mechanical reprogramming requires a secondary mechanism to latch the memory, such as mechanosensitive adherens junction reinforcement (e.g. E-cadherin clustering) or extracellular matrix remodeling. Our main results phenomenologically capture this latching mechanism by operating in the limit of slow tension relaxation. To confirm that the observed phenomena are indeed contingent upon this memory, we analyzed the effect of finite tension relaxation timescale on the tissue’s adaptive response (Section S11). We find that memory-based effects, including the change in shear modulus (ΔG), the cooperative long-range response (ΔΛ), and the change in Poisson ratio (Δν), are all diminished when tension relaxation is fast compared to junction remodeling rate ($\tau_{\Lambda }^{-1}\gg (k\sqrt{A_{0}})/\Lambda_{0}$), where *k* denotes the strain-dependent remodeling rate ($k=k_{C}$ for contractile strains and $k=k_{E}$ for extensional strains). Only when tension relaxation is slow does the tissue retain a significant, persistent memory and exhibit programmed mechanical changes. The rapid erasure of $\Lambda_{ij}$ eliminates all history-dependent mechanical responses, confirming that stable adaptation requires a mechanical latch that outlasts transient cytoskeletal turnover.

These behaviors can be probed using optogenetic myosin activation tests and Poisson ratio measurements under cyclic training (21, 26, 58, 59). Cyclic loading is known to affect epithelial mechanics: compared with constant tension, it prolongs monolayer lifetime (59). Whether it changes stiffness or Poisson ratio has not been measured. The model predicts that, for $k_{C}>k_{E}$ and strains above the junctional threshold, the Poisson ratio decreases by an amount that grows with strain amplitude and fades over the tension–relaxation time once loading stops. The auxetic state is thus a trained state and not a baseline, and the trained auxetic transition is a prediction of the model that is yet to be tested in experiments.

Many epithelia are loaded cyclically during normal function, as in the gut during peristalsis and the airway during breathing (59). In such tissues, tension remodeling would match stiffness to recent loading such that small strains are ignored and large repeated strains stiffen the tissue for $k_{C}>k_{E}$. The stiffening is lost once loading stops and tension relaxes. Loading by sliding shear, as on the cornea, was not simulated in this study. The remodeling machinery can be perturbed genetically. For instance, the loss of p120-catenin abolishes the mechanosensitivity of E-cadherin turnover and lowers tissue viscosity in the *Drosophila* wing (25). If remodeling chronically outpaces relaxation, stiffness accumulates and does not reset. In our model, this is prevented by weaker remodeling or faster relaxation. In this view, the capacity to learn is adaptive, and the failure to forget is a candidate mechanical route to pathological stiffening such as fibrosis.

## Supplementary Material

pgag367_Supplementary_Data

## Data Availability

This is a theoretical and computational study, and no experimental data were generated. The simulation code is available at https://github.com/BanerjeeLab/LearningTissue.

## References

[1] Hexner D, Pashine N, Liu AJ, Nagel SR. 2020. Effect of directed aging on nonlinear elasticity and memory formation in a material. Phys Rev Res. 2:043231.

[2] Jaeger HM, Murugan A, Nagel SR. 2024. Training physical matter to matter. Soft Matter. 20:6695–6701. 10.1039/d4sm00629a 39140794

[3] Keim NC, Paulsen JD, Zeravcic Z, Sastry S, Nagel SR. 2019. Memory formation in matter. Rev Mod Phys. 91:035002.

[4] Lindeman CW, Hagh VF, Ip CI, Nagel SR. 2023. Competition between energy and dynamics in memory formation. Phys Rev Lett. 130:197201. 10.1103/PhysRevLett.130.197201 37243648

[5] Mandal R, Tapias D, Sollich P. 2021. Memory in non-monotonic stress response of an athermal disordered solid. Phys Rev Res. 3:043153.

[6] Pashine N, Hexner D, Liu AJ, Nagel SR. 2019. Directed aging, memory, and nature’s greed. Sci Adv. 5:eaax4215. 10.1126/sciadv.aax4215 PMC6989340 32064313

[7] Shohat D, Hexner D, Lahini Y. 2022. Memory from coupled instabilities in unfolded crumpled sheets. Proc Natl Acad Sci U S A. 119:e2200028119. 10.1073/pnas.2200028119 PMC9282240 35867743

[8] Sudreau I, Manneville S, Servel M, Divoux T. 2022. Shear-induced memory effects in boehmite gels. J Rheol. 66:91–104.

[9] Fiocco D, Foffi G, Sastry S. 2013. Oscillatory athermal quasistatic deformation of a model glass. Phys Rev E. 88:020301. 10.1103/PhysRevE.88.020301 24032763

[10] Paulsen JD, Keim NC. 2025. Mechanical memories in solids, from disorder to design. Annu Rev Condens Matter Phys. 16:61–81.

[11] Regev I, Lookman T, Reichhardt C. 2013. Onset of irreversibility and chaos in amorphous solids under periodic shear. Phys Rev E. 88:062401. 10.1103/PhysRevE.88.062401 24483455

[12] Altman LE, Stern M, Liu AJ, Durian DJ. 2024. Experimental demonstration of coupled learning in elastic networks. Phys Rev Appl. 22:024053.

[13] Anisetti VR, Kandala A, Scellier B, Schwarz JM. 2024. Frequency propagation: multimechanism learning in nonlinear physical networks. Neural Comput. 36:596–620. 10.1162/neco_a_01648 38457749

[14] Banerjee DS , et al 2026. Learning via mechanosensitivity and activity in cytoskeletal networks. PRX Life. 4:013001.

[15] Dillavou S, Stern M, Liu AJ, Durian DJ. 2022. Demonstration of decentralized physics-driven learning. Phys Rev Appl. 18:014040. 10.1063/5.0084631 35428388

[16] Guzman M, Martins F, Stern M, Liu AJ. 2025. Microscopic imprints of learned solutions in tunable networks. Phys Rev X. 15:031056.

[17] Scellier B, Bengio Y. 2017. Equilibrium propagation: bridging the gap between energy-based models and backpropagation. Front Comput Neurosci. 11:24. 10.3389/fncom.2017.00024 PMC5415673 28522969

[18] Stern M, Hexner D, Rocks JW, Liu AJ. 2021. Supervised learning in physical networks: from machine learning to learning machines. Phys Rev X. 11:021045.

[19] Stern M, Murugan A. 2023. Learning without neurons in physical systems. Annu Rev Condens Matter Phys. 14:417–441.

[20] Hexner D, Liu AJ, Nagel SR. 2020. Periodic training of creeping solids. Proc Natl Acad Sci U S A. 117:31690–31695. 10.1073/pnas.1922847117 PMC7749283 33257582

[21] Cavanaugh KE , et al 2022. Force-dependent intercellular adhesion strengthening underlies asymmetric adherens junction contraction. Curr Biol. 32:1986–2000.e5. 10.1016/j.cub.2022.03.024 PMC9123775 35381185

[22] Devany J, Sussman DM, Yamamoto T, Manning ML, Gardel ML. 2021. Cell cycle-dependent active stress drives epithelia remodeling. Proc Natl Acad Sci U S A. 118:e1917853118. 10.1073/pnas.1917853118 PMC7958291 33649197

[23] Heisenberg C-P, Bellaïche Y. 2013. Forces in tissue morphogenesis and patterning. Cell. 153:948–962. 10.1016/j.cell.2013.05.008 23706734

[24] Herrera-Perez RM, Cupo C, Allan C, Dagle AB, Kasza KE. 2023. Tissue flows are tuned by actomyosin-dependent mechanics in developing embryos. PRX Life. 1:013004. 10.1103/PRXLife.1.013004 PMC11086709 38736460

[25] Iyer KV, Piscitello-Gómez R, Paijmans J, Jülicher F, Eaton S. 2019. Epithelial viscoelasticity is regulated by mechanosensitive E-cadherin turnover. Curr Biol. 29:578–591.e5. 10.1016/j.cub.2019.01.021 30744966

[26] Khalilgharibi N , et al 2019. Stress relaxation in epithelial monolayers is controlled by the actomyosin cortex. Nat Phys. 15:839–847. 10.1038/s41567-019-0516-6 PMC7116713 33569083

[27] Lecuit T, Lenne P-F. 2007. Cell surface mechanics and the control of cell shape, tissue patterns and morphogenesis. Nat Rev Mol Cell Biol. 8:633–644. 10.1038/nrm2222 17643125

[28] Shraiman BI . 2005. Mechanical feedback as a possible regulator of tissue growth. Proc Natl Acad Sci U S A. 102:3318–3323. 10.1073/pnas.0404782102 PMC552900 15728365

[29] Wyatt TPJ , et al 2020. Actomyosin controls planarity and folding of epithelia in response to compression. Nat Mater. 19:109–117. 10.1038/s41563-019-0461-x 31451778

[30] Nishizawa K, Lin S-Z, Chardès C, Rupprecht J-F, Lenne P-F. 2023. Two-point optical manipulation reveals mechanosensitive remodeling of cell–cell contacts in vivo. Proc Natl Acad Sci U S A. 120:e2212389120. 10.1073/pnas.2212389120 PMC10068846 36947511

[31] Bi D, Lopez JH, Schwarz JM, Manning ML. 2015. A density-independent rigidity transition in biological tissues. Nat Phys. 11:1074–1079.

[32] Farhadifar R, Röper J-C, Aigouy B, Eaton S, Jülicher F. 2007. The influence of cell mechanics, cell-cell interactions, and proliferation on epithelial packing. Curr Biol. 17:2095–2104. 10.1016/j.cub.2007.11.049 18082406

[33] Tong S, Singh NK, Sknepnek R, Košmrlj A. 2022. Linear viscoelastic properties of the vertex model for epithelial tissues. PLoS Comput Biol. 18:e1010135. 10.1371/journal.pcbi.1010135 PMC9159552 35587514

[34] Cavanaugh KE, Staddon MF, Munro E, Banerjee S, Gardel ML. 2020. RhoA mediates epithelial cell shape changes via mechanosensitive endocytosis. Dev Cell. 52:152–166.e5. 10.1016/j.devcel.2019.12.002 PMC7565439 31883774

[35] Staddon MF, Cavanaugh KE, Munro EM, Gardel ML, Banerjee S. 2019. Mechanosensitive junction remodeling promotes robust epithelial morphogenesis. Biophys J. 117:1739–1750. 10.1016/j.bpj.2019.09.027 PMC6838884 31635790

[36] Arzash S, Liu AJ, Manning ML. 2026. Epithelial convergent extension as a tuning process. Proc Natl Acad Sci U S A. 123:e2532099123. 10.1073/pnas.2532099123 PMC13552903 42673465

[37] Arzash S, Tah I, Liu AJ, Manning ML. 2025. Rigidity of epithelial tissues as a double optimization problem. Phys Rev Res. 7:013157.

[38] Pérez-Verdugo F, Banerjee S. 2023. Tension remodeling regulates topological transitions in epithelial tissues. PRX Life. 1:023006. 10.1103/prxlife.1.023006 PMC11500814 39450340

[39] Pérez-Verdugo F, Banks S, Banerjee S. 2024. Excitable dynamics driven by mechanical feedback in biological tissues. Commun Phys. 7:167. 10.1038/s42005-024-01661-2 PMC12395394 40893445

[40] Alt S, Ganguly P, Salbreux G. 2017. Vertex models: from cell mechanics to tissue morphogenesis. Philos Trans R Soc B: Biol Sci. 372:20150520. 10.1098/rstb.2015.0520 PMC5379026 28348254

[41] Fletcher AG, Osterfield M, Baker RE, Shvartsman SY. 2014. Vertex models of epithelial morphogenesis. Biophys J. 106:2291–2304. 10.1016/j.bpj.2013.11.4498 PMC4052277 24896108

[42] Nagai T, Honda H. 2001. A dynamic cell model for the formation of epithelial tissues. Philos Mag B. 81:699–719.

[43] Sussman DM . 2017. cellGPU: massively parallel simulations of dynamic vertex models. Comput Phys Commun. 219:400–406.

[44] Bitzek E, Koskinen P, Gähler F, Moseler M, Gumbsch P. 2006. Structural relaxation made simple. Phys Rev Lett. 97:170201. 10.1103/PhysRevLett.97.170201 17155444

[45] Martin AC . 2010. Pulsation and stabilization: contractile forces that underlie morphogenesis. Dev Biol. 341:114–125. 10.1016/j.ydbio.2009.10.031 19874815

[46] Martin AC, Kaschube M, Wieschaus EF. 2009. Pulsed contractions of an actin–myosin network drive apical constriction. Nature. 457:495–499. 10.1038/nature07522 PMC2822715 19029882

[47] Michaux JB, Robin FB, McFadden WM, Munro EM. 2018. Excitable RhoA dynamics drive pulsed contractions in the early *C. elegans* embryo. J Cell Biol. 217:4230–4252. 10.1083/jcb.201806161 PMC6279378 30275107

[48] Munro E, Nance J, Priess JR. 2004. Cortical flows powered by asymmetrical contraction transport par proteins to establish and maintain anterior-posterior polarity in the early *C. elegans* embryo. Dev Cell. 7:413–424. 10.1016/j.devcel.2004.08.001 15363415

[49] Staddon MF, Munro EM, Banerjee S. 2022. Pulsatile contractions and pattern formation in excitable actomyosin cortex. PLoS Comput Biol. 18:e1009981. 10.1371/journal.pcbi.1009981 PMC9000090 35353813

[50] Bouton ME . Learning and behavior: a contemporary synthesis. Sinauer Associates, 2007.

[51] Cavanaugh KE, Staddon MF, Banerjee S, Gardel ML. 2020. Adaptive viscoelasticity of epithelial cell junctions: from models to methods. Curr Opin Genet Dev. 63:86–94. 10.1016/j.gde.2020.05.018 PMC7483996 32604032

[52] Damavandi OK, Arzash S, Lawson-Keister E, Manning ML. 2025. Universality in the mechanical behavior of vertex models for biological tissues. PRX Life. 3:033001.

[53] Merkel M, Manning ML. 2018. A geometrically controlled rigidity transition in a model for confluent 3D tissues. New J Phys. 20:022002.

[54] Eshelby JD . 1957. The determination of the elastic field of an ellipsoidal inclusion, and related problems. Proc R Soc Lond A Math Phys Sci. 241:376–396.

[55] Bertoldi K . 2017. Harnessing instabilities to design tunable architected cellular materials. Annu Rev Mater Res. 47:51–61.

[56] Lakes R . 1987. Foam structures with a negative Poisson’s ratio. Science. 235:1038–1040. 10.1126/science.235.4792.1038 17782252

[57] Reid DR , et al 2018. Auxetic metamaterials from disordered networks. Proc Natl Acad Sci U S A. 115:E1384–E1390. 10.1073/pnas.1717442115 PMC5816187 29382758

[58] Harris AR , et al 2012. Characterizing the mechanics of cultured cell monolayers. Proc Natl Acad Sci U S A. 109:16449–16454. 10.1073/pnas.1213301109 PMC3478631 22991459

[59] Papafilippou E, Bonfanti A, Charras G, Kabla A. 2026. Emergent intercellular junction stability during cyclic tissue loading. Biophys J. 125:4585–4601. 10.1016/j.bpj.2026.06.038 42400154

