# Supplementary material for: Learning epithelial elasticity via local tension remodeling
Source: PNAS Nexus. 2026 Oct 25;5(11):pgag367. doi: 10.1093/pnasnexus/pgag367 (PMC13741241; doi:10.1093/pnasnexus/pgag367)
Supplement: Supplementary file 1 [file pgag367_supplementary_data.zip › PNASNEXUS-PNASNEXUS-2026-01287-TR-s04.pdf]

Supporting Information

Learning Epithelial Elasticity via Local Tension Remodeling

Sadjad Arzash and Shiladitya Banerjee*

School of Physics, Georgia Institute of Technology, Atlanta, GA 30332, USA

*To whom correspondence should be addressed: sbanerjee347@gatech.edu

This file includes:

Supplementary text, Sections S1 to S12
Figs. S1 to S12
Legends for Movies S1 to S3
References

Other supplementary material for this manuscript:

Movies S1 to S3

Contents

S1 Effective viscoelastic kernel from tension remodeling 2
S2 Extension of an edge under contractile signals 4
S3 Effect of active contractility period 5
S4 Finite-size effects 6
S5 Continuum theory of cooperative mechanical memory 7
S6 Structural features 10
S7 Effect of T1 transitions on trained mechanical response 11
S8 Minimal mean-field model for auxetic response 13
S9 Comparison between trained tissues and static high-tension vertex models 14
S10 Non-affine character of the trained auxetic response 17
S11 Effect of tension relaxation 18
S12 Effect of stochastic junctional tension fluctuations 20
S13 Supplementary Movies 21

S1 Effective viscoelastic kernel from tension remodeling

We derive the memory kernel $\mathcal{K}(\tau)$ that governs the history-dependent mechanical response of the adaptive vertex model. The tension-remodeling dynamics for each edge ij are:

$$\frac{\mathrm{d}\Lambda_{ij}}{\mathrm{d}t} = -k(\varepsilon_{ij})\,(\ell_{ij} - \ell_{ij}^0) - \frac{1}{\tau_\Lambda}\,(\Lambda_{ij} - \Lambda_0)\,, \tag{S1}$$

$$\frac{\mathrm{d}\ell_{ij}^0}{\mathrm{d}t} = k_L\,(\ell_{ij} - \ell_{ij}^0)\,, \tag{S2}$$

where $k(\varepsilon_{ij}) = k_C$ for $\varepsilon < -\varepsilon_c$, $k(\varepsilon_{ij}) = k_E$ for $\varepsilon > \varepsilon_c$, and zero otherwise.

Consider an edge ij in the remodeling-active regime, where $|\varepsilon_{ij}| > \varepsilon_c$ and the remodeling rate takes a constant value $\bar{k}$ ($\bar{k} = k_C$ for $\varepsilon < -\varepsilon_c$, $\bar{k} = k_E$ for $\varepsilon > \varepsilon_c$). Define perturbations around a uniform reference state with tension Λ_0, edge length ℓ_0, and rest length $\ell_0^0 = \ell_0$:

$$\delta\Lambda_{ij}(t) = \Lambda_{ij}(t) - \Lambda_0\,, \tag{S3}$$

$$\delta\ell_{ij}(t) = \ell_{ij}(t) - \ell_0\,, \tag{S4}$$

$$\delta\ell_{ij}^0(t) = \ell_{ij}^0(t) - \ell_0\,. \tag{S5}$$

We treat $\delta\ell_{ij}(t)$ as a prescribed input, determined by the fast vertex dynamics under external driving. This separation of timescales is the central assumption: the vertices relax quickly to mechanical equilibrium at each instant, while the tensions and rest lengths evolve slowly.

Linearizing (S1) and (S2):

$$\frac{\mathrm{d}\,\delta\Lambda_{ij}}{\mathrm{d}t} = -\bar{k}\,(\delta\ell_{ij} - \delta\ell_{ij}^0) - \frac{1}{\tau_\Lambda}\,\delta\Lambda_{ij}\,, \tag{S6}$$

$$\frac{\mathrm{d}\,\delta\ell_{ij}^0}{\mathrm{d}t} = k_L\,(\delta\ell_{ij} - \delta\ell_{ij}^0)\,. \tag{S7}$$

These are two coupled linear ODEs driven by $\delta\ell_{ij}(t)$.

Define the strain mismatch $s_{ij}(t) = \delta\ell_{ij}(t) - \delta\ell_{ij}^0(t)$. From (S7):

$$\frac{\mathrm{d}s_{ij}}{\mathrm{d}t} = \dot{\delta\ell}_{ij} - k_L\,s_{ij}\,. \tag{S8}$$

This is a first-order linear ODE with solution

$$s_{ij}(t) = \int_{-\infty}^{t} e^{-k_L(t-t')}\,\dot{\delta\ell}_{ij}(t')\,\mathrm{d}t'\,. \tag{S9}$$

Substituting into (S6):

$$\frac{\mathrm{d}\,\delta\Lambda_{ij}}{\mathrm{d}t} + \frac{\delta\Lambda_{ij}}{\tau_\Lambda} = -\bar{k}\int_{-\infty}^{t} e^{-k_L(t-t')}\,\dot{\delta\ell}_{ij}(t')\,\mathrm{d}t'\,. \tag{S10}$$

Solving this forced first-order ODE by variation of parameters:

$$\delta\Lambda_{ij}(t) = -\bar{k}\int_{-\infty}^{t} \dot{\delta\ell}_{ij}(t')\,\underbrace{\int_{t'}^{t} e^{-(t-t'')/\tau_\Lambda}\,e^{-k_L(t''-t')}\,\mathrm{d}t''}_{\equiv\,\mathcal{K}(t-t')}\,\mathrm{d}t'\,. \tag{S11}$$

Evaluating the inner integral (with $\tau = t - t'$ and substitution $u = t'' - t'$):

$$\begin{aligned}
\mathcal{K}(\tau) &= \int_0^\tau e^{-(\tau-u)/\tau_\Lambda}\, e^{-k_L u}\,\mathrm{d}u \\
&= e^{-\tau/\tau_\Lambda} \int_0^\tau e^{-(k_L - 1/\tau_\Lambda)\,u}\,\mathrm{d}u \\
&= \frac{e^{-\tau/\tau_\Lambda} - e^{-k_L \tau}}{k_L - 1/\tau_\Lambda}\,.
\end{aligned} \tag{S12}$$

This is valid for $k_L \neq 1/\tau_\Lambda$. In the degenerate case $k_L = 1/\tau_\Lambda$, $\mathcal{K}(\tau) = \tau\, e^{-k_L \tau}$.

The tension response is therefore a convolution:

$$\delta\Lambda_{ij}(t) = -\bar{k} \int_{-\infty}^{t} \mathcal{K}(t - t')\, \delta\dot{\ell}_{ij}(t')\,\mathrm{d}t'\,. \tag{S13}$$

Properties of the memory kernel

Vanishing at $\tau = 0$. $\mathcal{K}(0) = 0$. A sudden deformation produces no instantaneous tension change through the slow variables. The immediate elastic response comes entirely from the contractility term $\Gamma_a \ell^2$ in the energy.

Two-timescale structure. $\mathcal{K}(\tau)$ rises on the timescale k_L^{-1} (rest-length relaxation) and decays on the timescale τ_Λ (tension relaxation). The peak occurs at

$$\tau_{\text{peak}} = \frac{\ln(k_L \tau_\Lambda)}{k_L - 1/\tau_\Lambda}\,. \tag{S14}$$

Permanent memory ($\tau_\Lambda \to \infty$). In the limit of slow tension relaxation:

$$\mathcal{K}(\tau) \xrightarrow{\tau_\Lambda \to \infty} \frac{1 - e^{-k_L \tau}}{k_L}\,, \tag{S15}$$

which saturates at $1/k_L$ for $\tau \gg k_L^{-1}$. After a step deformation $\delta\ell_{ij}(t) = \delta\ell_0\,\Theta(t)$, the tension reaches a permanent steady-state change:

$$\delta\Lambda_\infty = -\bar{k}\,\delta\ell_0\,\mathcal{K}(\infty) = -\frac{\bar{k}}{k_L}\,\delta\ell_0 = -\tilde{k}\,\delta\ell_0\,, \tag{S16}$$

where $\tilde{k} = \bar{k}/k_L$ is the dimensionless remodeling rate ($\tilde{k}_C$ or $\tilde{k}_E$). The dimensionless remodeling rate thus directly sets the magnitude of the persistent tension change per unit deformation, providing the analytical origin of the permanent stiffness changes observed in simulations.

Memory erasure (τ_Λ finite). When τ_Λ is finite, the kernel decays exponentially at long times: $\mathcal{K}(\tau) \sim e^{-\tau/\tau_\Lambda}$ for $\tau \gg k_L^{-1}$. A step deformation now produces a transient tension change that builds up on timescale k_L^{-1} and relaxes back to zero on timescale τ_Λ. The tissue retains memory only for times $t \lesssim \tau_\Lambda$ after the stimulus. In the fast-relaxation limit ($\tau_\Lambda \ll k_L^{-1}$), the kernel becomes narrow and weak: $\mathcal{K}(\tau) \approx \tau_\Lambda\, e^{-k_L \tau}$, and the memory contribution is negligible.

Passive limit. When $\bar{k} = 0$ (no remodeling), $\delta\Lambda_{ij}(t) = 0$ identically: there is no history-dependent contribution, and the edge responds as a purely elastic element with force $\delta f_{ij} = 2\Gamma_a\,\delta\ell_{ij}$. This is the formal distinction between the adaptive model and a static vertex model.

Fast rest-length relaxation ($k_L \to \infty$ with $\tilde{k} = \bar{k}/k_L$ fixed). The kernel reduces to a single exponential, $\bar{k}\,\mathcal{K}(\tau) \to \tilde{k}\,e^{-\tau/\tau_\Lambda}$, and the constitutive relation becomes that of a spring in parallel with a Maxwell element of stiffness $-\tilde{k}$ and relaxation time τ_Λ. After a step strain, the force recovers to the purely elastic value $2\Gamma_a\,\delta\ell_0$, the tissue fully forgets, and no permanent memory is retained.

The total edge force perturbation is $\delta f_{ij} = \delta\Lambda_{ij} + 2\Gamma_a\,\delta\ell_{ij}$ (from $T_{ij} = \Lambda_{ij} + 2\Gamma_a \ell_{ij}$). Substituting (S13):

$$\boxed{\delta f_{ij}(t) = 2\Gamma_a\,\delta\ell_{ij}(t) - \bar{k}\int_{-\infty}^{t} \mathcal{K}(t-t')\,\dot{\delta\ell}_{ij}(t')\,\mathrm{d}t' \,.} \tag{S17}$$

This is a linear viscoelastic constitutive relation. The instantaneous elastic modulus is $2\Gamma_a$ (from the contractility term in the energy), and the memory contribution from the slow remodeling variables produces history-dependent stress relaxation and energy dissipation.

The above equation places the adaptive tissue in the generalized-Maxwell family of viscoelastic solids, with two internal relaxation timescales k_L^{-1} and τ_Λ producing the biexponential kernel $\mathcal{K}(\tau)$. Unlike a Kelvin–Voigt material, whose damping is proportional to the instantaneous strain rate, the dissipation here is non-local in time: a convolution of the strain rate against $\mathcal{K}$. More fundamentally, the kernel is conditional, vanishing whenever the local strain stays below the remodeling threshold ε_c. The tissue is therefore elastic under small perturbations and viscoelastic with memory only when driven above threshold. This threshold-gated rheology distinguishes it from any passive viscoelastic material and constitutes the analytical signature of learning matter.

Stress relaxation

Consider a step strain applied at $t = 0$: $\delta\ell_{ij}(t) = \delta\ell_0\,\Theta(t)$, where Θ is the Heaviside function. Then $\dot{\delta\ell}_{ij} = \delta\ell_0\,\delta(t)$, and:

$$\delta f_{ij}(t) = \delta\ell_0\big[2\Gamma_a - \bar{k}\,\mathcal{K}(t)\big]\,, \qquad t > 0\,. \tag{S18}$$

The edge force starts at the instantaneous elastic value $2\Gamma_a\,\delta\ell_0$ (since $\mathcal{K}(0) = 0$) and then relaxes as $\mathcal{K}(t)$ builds up.

In the limit $\tau_\Lambda \to \infty$, the long-time force is:

$$\delta f_\infty = \delta\ell_0\big[2\Gamma_a - \tilde{k}\big]\,, \tag{S19}$$

reduced relative to the instantaneous value by the dimensionless remodeling rate $\tilde{k}$. This slow relaxation is a direct prediction for additional relaxation in the trained tissue beyond the fast overdamped vertex dynamics, while the static high-tension control reaches its plateau immediately.

For finite τ_Λ, the force relaxes to the plateau of Eq. (S19) and then *recovers* back toward $2\Gamma_a\,\delta\ell_0$ on timescale τ_Λ, as the tension relaxation erases the remodeling:

$$\delta f_{ij}(t) \approx \delta\ell_0\left[2\Gamma_a - \tilde{k}\,e^{-t/\tau_\Lambda}\right]\,, \qquad t \gg k_L^{-1}\,. \tag{S20}$$

The tissue therefore exhibits three temporal regimes: (i) instantaneous elastic response ($t \ll k_L^{-1}$), (ii) slow relaxation from remodeling ($k_L^{-1} \ll t \ll \tau_\Lambda$), and (iii) recovery from tension relaxation ($t \gg \tau_\Lambda$).

S2 Extension of an edge under contractile signals

In the main text, we showed that when $\tilde{k}_E > \tilde{k}_C$, an edge subjected to pulsatile contractile signals can surprisingly undergo a net extension over time. Figure S1 illustrates this behavior. During each

contractile pulse, the edge initially shortens, as expected. However, in the relaxation phase that follows, the influence of neighboring edges places the junction in a strain regime where $\varepsilon > \varepsilon_c$. In this regime, the tension-remodeling rule reduces the edge tension. Repeated cycles of contraction and relaxation therefore drive a progressive decrease in tension, leading to a net extension of the edge despite the applied contractile signals.

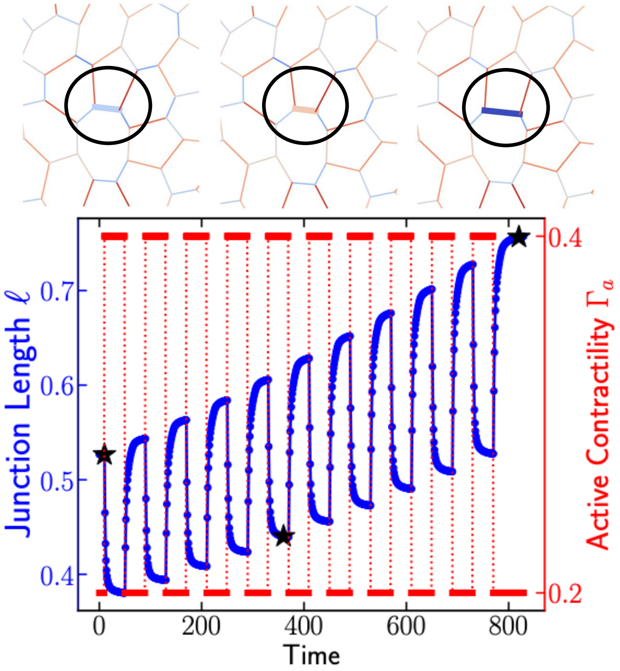

Fig. S1. Edge length (left, blue axis) and applied contractile signal (right, red axis) as functions of time in the regime $\tilde{k}_E > \tilde{k}_C$ (here $\tilde{k}_E = 0.2$ and $\tilde{k}_C = 0$). The top row shows consecutive snapshots of the edge corresponding to the time points marked with black stars in the main plot. Here, the junction-level critical strain is $\varepsilon_c = 0.1$.

S3 Effect of active contractility period

The duration of the active contractile pulse strongly influences the edge response. Figure S2b shows the net change in edge length as a function of the contractility period $\mathcal{T}$. For very short periods, the edge has insufficient time to remodel, resulting in negligible length change. As $\mathcal{T}$ increases, the accumulated remodeling leads to a monotonic increase in length change, which eventually saturates to a plateau. This has been observed in experiments on epithelial junctions (1).

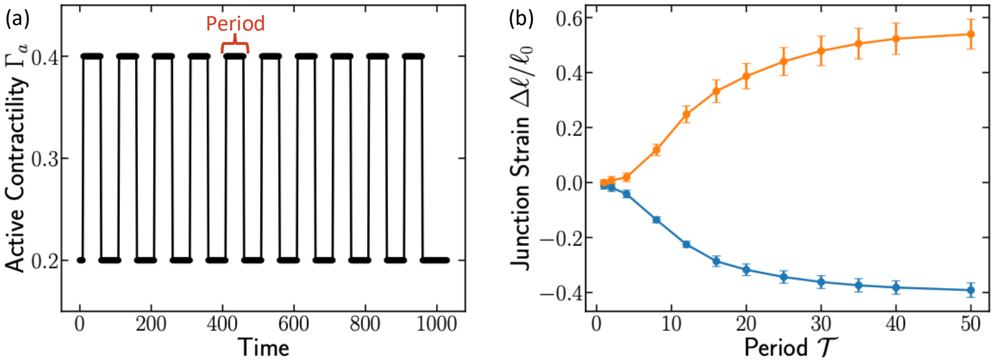

Fig. S2. (a) Pulsatile contractility signal alternating between a low value $\Gamma_a = 0.2$ and a high value $\Gamma_a = 0.4$. The period of this signal controls the extent of junctional remodeling. (b) Final change in junction length after applying the signal in (a) for different periods $\mathcal{T}$. The orange curve corresponds to the regime $\tilde{k}_E > \tilde{k}_C$ (here $\tilde{k}_E = 0.2$, $\tilde{k}_C = 0$), where edges exhibit net extension. The blue curve shows the regime $\tilde{k}_C > \tilde{k}_E$ (here $\tilde{k}_C = 0.2$, $\tilde{k}_E = 0$), where edges contract under repeated pulses.

S4 Finite-size effects

In the main text, we show that localized pulsatile contractility can reprogram the global shear modulus of the tissue. We also show that the magnitude of the shear modulus change depends on the size of the activated region, suggesting that the response is controlled by the size of the active domain. To determine whether this effect persists in finite tissues, we performed additional simulations in which the total number of cells was varied while keeping the relative size of the activated region fixed.

For each tissue size, we initialized a periodic tissue with N cells and applied five pulsatile contractile activations to a circular region at the center of the tissue. The activation radius was scaled with system size so that $\frac{R}{\sqrt{N}} = \frac{1}{6}$. Thus, the activated region occupies the same relative size in each system. Each pulse had duration 40. The simulations shown here were performed in the extension-dominated remodeling regime, with $\tilde{k}_C = 0$ and $\tilde{k}_E = 0.25$. After the activation protocol, the tissue was relaxed to a force-balanced state with the remodeled tensions held fixed, and the shear modulus was computed using the procedure described in the main text.

Figure S3 shows the normalized change in shear modulus, $\Delta G/G_0$, as a function of the total number of cells. Small tissues show clear finite-size effects, as expected when the activated region and the surrounding elastic medium are both represented by only a limited number of cells. As N increases, however, $\Delta G/G_0$ approaches a plateau. This saturation indicates that the modulus change converges to a well-defined response. Although the data shown here correspond to $\tilde{k}_E > \tilde{k}_C$, the same finite-size behavior is observed across the parameter regimes studied.

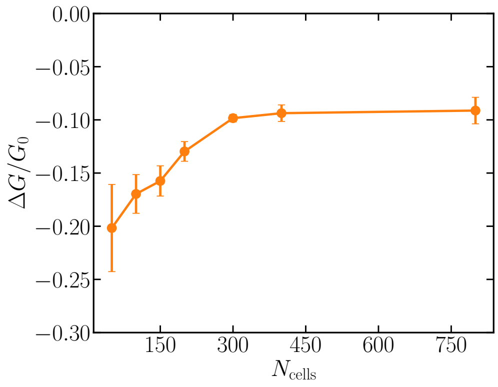

Fig. S3. Finite-size dependence of contractility-induced modulus reprogramming. The normalized change in shear modulus, $\Delta G/G_0$, is plotted as a function of the total number of cells, N_{cells}, after five pulsatile contractile activations applied to a circular region with fixed relative radius $R/\sqrt{N} = 1/6$. The pulse duration is 40, with remodeling parameters $\tilde{k}_C = 0$ and $\tilde{k}_E = 0.25$. The response shows finite-size effects for small tissues but approaches a plateau at larger N, indicating convergence to a bulk stiffness change.

S5 Continuum theory of cooperative mechanical memory

Here we derive a continuum description of the vertex model to explain long-range memory and cooperative tension enhancement in the tissue. The mechanical energy of the tissue is

$$E = \sum_{\alpha=1}^{N} K_A (A_\alpha - A_0)^2 + \sum_{\langle ij \rangle} \left[\Lambda_{ij}\, \ell_{ij} + \Gamma_a\, \ell_{ij}^2 \right]. \tag{S21}$$

We coarse-grain this energy functional to extract the effective elastic moduli of the tissue, solve for the tissue strain field in response to a local contraction within a finite periodic box, and derive the analytical expression for the cooperative tension enhancement in the tissue.

Effective elastic moduli – Consider the uniform reference state: all cell areas $A_\alpha = A_0$, all active tensions $\Lambda_{ij} = \Lambda_0$, all edge lengths $\ell_{ij} = \ell_0$, and total edge tension $T_0 = \Lambda_0 + 2\Gamma_a \ell_0$. We extract the 2D bulk and shear moduli by applying uniform deformations and expanding (S21) to second order. Under a uniform isotropic dilation $\mathbf{r}_i \to (1+\varepsilon)\mathbf{r}_i$, cell areas scale as $A_\alpha \to (1+2\varepsilon)A_0 + \mathcal{O}(\varepsilon^2)$ and edge lengths as $\ell_{ij} \to (1+\varepsilon)\ell_0$. Expanding (S21) to second order:

$$\Delta E = 4NK_A A_0^2\, \varepsilon^2 + N_e\, \Gamma_a\, \ell_0^2\, \varepsilon^2 + (\text{linear terms}), \tag{S22}$$

where N is the number of cells and N_e the total number of edges. The linear terms encode the residual prestress and vanish in force-balanced reference configurations. The 2D bulk modulus is defined by $\Delta E = \frac{1}{2} B\, \mathcal{A}\, (\operatorname{tr} \varepsilon)^2$ with total area $\mathcal{A} = NA_0$ and $\operatorname{tr}\varepsilon = 2\varepsilon$ in 2D, giving:

$$B = 2K_A A_0 + \frac{N_e}{2N} \frac{\Gamma_a\, \ell_0^2}{A_0}. \tag{S23}$$

For a Voronoi tiling, $N_e \approx 3N$, so $B = 2K_A A_0 + \frac{3\Gamma_a \ell_0^2}{2A_0}$. With the nondimensionalization $K_A = 1$, $A_0 = 1$: $B = 2 + \frac{3}{2}\Gamma_a \ell_0^2$.

Under simple shear ($x_i \to x_i + \gamma\, y_i$), cell areas are unchanged to linear order, so only the edge terms contribute. In the solid phase, the shear modulus scales as

$$G = c_G \frac{T_0}{\sqrt{A_0}}, \tag{S24}$$

where $T_0 = \Lambda_0 + 2\Gamma_a \ell_0$ is the total edge tension and $c_G = \mathcal{O}(1)$ is a geometric prefactor that depends on the specific tiling and the proximity to the rigidity transition. For a given reference tissue, G is computed from the Hessian of the energy at the reference state.

Eigenstress from the activated region – After Region 1 (a circular region of radius R) is activated and the tissue has fully relaxed, edges inside Region 1 carry permanently elevated active tensions $\Lambda_{ij} = \Lambda_0 + \delta\Lambda_{\rm src}$, where $\delta\Lambda_{\rm src} > 0$ is the net tension accumulated through the junctional ratcheting mechanism.

The coarse-grained stress perturbation inside Region 1 is obtained by summing over edge contributions to the Irving–Kirkwood stress tensor. For an edge with orientation $\hat{\mathbf{e}}_{ij} = (\cos\theta, \sin\theta)$ and tension increment $\delta\Lambda_{\rm src}$, the stress contribution is $\propto \delta\Lambda_{\rm src}\, \ell_0\, \hat{e}_{ij,m}\, \hat{e}_{ij,n}$. Averaging over the random orientations of the many edges inside Region 1 ($\langle \hat{e}_m \hat{e}_n \rangle_\theta = \frac{1}{2}\delta_{mn}$ for an isotropic network), the eigenstress is

$$\delta\sigma^{\rm src}_{mn}(\mathbf{r}) = p_0\, \delta_{mn}\, \Theta_R(\mathbf{r}) \,, \tag{S25}$$

where $\Theta_R(\mathbf{r})$ is the indicator function of Region 1, and the eigenstress amplitude is

$$p_0 = \frac{n_e\, \delta\Lambda_{\rm src}\, \ell_0}{2} \,, \tag{S26}$$

with $n_e = N_e/(NA_0)$ the edge number density. The eigenstress is isotropic (proportional to δ_{mn}), reflecting the statistical isotropy of the disordered network inside the activation region.

Navier equation and Fourier-space solution – The displacement field $\mathbf{u}(\mathbf{r})$ satisfies the 2D Navier equation with a body force $\mathbf{f} = -p_0 \nabla\Theta_R$:

$$(\lambda + \mu)\, \nabla(\nabla\cdot\mathbf{u}) + \mu\, \nabla^2\mathbf{u} = -p_0\, \nabla\Theta_R \,, \tag{S27}$$

where λ and μ are the 2D Lamé parameters ($B = \lambda + \mu$, $G = \mu$).

We solve (S27) in Fourier space in the $L \times L$ periodic box, with wavevectors $\mathbf{q} = (2\pi/L)(n_x, n_y)$, $n_x, n_y \in \mathbb{Z}$. The Fourier transform of the indicator function is

$$\hat{\Theta}_R(\mathbf{q}) = \frac{1}{L^2} \int_{|\mathbf{r}|<R} e^{-i\mathbf{q}\cdot\mathbf{r}}\, \mathrm{d}^2 r = \phi\, \frac{2J_1(qR)}{qR} \,, \tag{S28}$$

where $\phi = \pi R^2/L^2$ and J_1 is the Bessel function of the first kind. At $\mathbf{q} = \mathbf{0}$: $\hat{\Theta}_R(\mathbf{0}) = \phi$.

Since the source $p_0 \nabla\Theta_R$ is purely longitudinal (a gradient of a scalar), the displacement field is irrotational. Taking the Fourier transform ($\partial_n \to iq_n$) of (S27), the second spatial derivatives produce factors of $i^2 = -1$ on the left-hand side, giving:

$$-\big[(\lambda + \mu) q_m q_n + \mu q^2 \delta_{mn}\big]\, \hat{u}_n = -ip_0\, q_m\, \hat{\Theta}_R \,. \tag{S29}$$

Multiplying both sides by -1:

$$\big[(\lambda + \mu) q_m q_n + \mu q^2 \delta_{mn}\big]\, \hat{u}_n = ip_0\, q_m\, \hat{\Theta}_R \,. \tag{S30}$$

The right-hand side is proportional to q_m (purely longitudinal), so the transverse component $\hat{u}_T$ is source-free and vanishes: $\hat{u}_T = 0$. To extract the dilation, we dot both sides of (S30) with iq_m:

$$\text{LHS:}\quad iq_m \big[(\lambda + \mu) q_m q_n + \mu q^2 \delta_{mn}\big]\, \hat{u}_n = (\lambda + 2\mu)\, q^2\, \widehat{\nabla\cdot\mathbf{u}} \,,$$
$$\text{RHS:}\quad iq_m \cdot ip_0\, q_m\, \hat{\Theta}_R = i^2\, p_0\, q^2\, \hat{\Theta}_R = -p_0\, q^2\, \hat{\Theta}_R \,.$$

Equating the two sides and dividing by q^2 gives the dilation in Fourier space, $\widehat{\nabla\cdot\mathbf{u}}(\mathbf{q}) = iq\hat{u}_L$:

$$\widehat{\nabla\cdot\mathbf{u}}(\mathbf{q}) = -\frac{p_0}{\lambda + 2\mu}\,\hat{\Theta}_R(\mathbf{q}) = -\frac{p_0}{B+G}\,\hat{\Theta}_R(\mathbf{q})\,, \qquad \mathbf{q} \neq \mathbf{0}\,. \tag{S31}$$

Here $\lambda + 2\mu = B + G$ is the 2D longitudinal modulus.

The $\mathbf{q} = \mathbf{0}$ mode is determined by the boundary conditions. In the fixed-box simulations, total area is conserved:

$$\widehat{\nabla\cdot\mathbf{u}}(\mathbf{0}) = 0\,. \tag{S32}$$

Dilation field – Transforming (S31) back to real space using the constraint (S32):

$$\begin{aligned}\nabla\cdot\mathbf{u}(\mathbf{r}) &= \sum_{\mathbf{q}\neq\mathbf{0}} \widehat{\nabla\cdot\mathbf{u}}(\mathbf{q})\,e^{i\mathbf{q}\cdot\mathbf{r}} \\ &= -\frac{p_0}{B+G}\left[\sum_{\mathbf{q}} \hat{\Theta}_R(\mathbf{q})\,e^{i\mathbf{q}\cdot\mathbf{r}} - \hat{\Theta}_R(\mathbf{0})\right] \\ &= -\frac{p_0}{B+G}\left[\Theta_R(\mathbf{r}) - \phi\right]. \end{aligned} \tag{S33}$$

This gives the central result:

$$\nabla\cdot\mathbf{u}(\mathbf{r}) = \begin{cases} -\dfrac{p_0\,(1-\phi)}{B+G}\,, & |\mathbf{r}| < R\,, \\ +\dfrac{p_0\,\phi}{B+G}\,, & |\mathbf{r}| > R\,. \end{cases} \tag{S34}$$

The dilation field outside Region 1 is spatially uniform and positive, implying that the tissue is uniformly dilated by an amount $p_0\phi/(B+G)$, independent of position. The eigenstress inside Region 1 compresses the tissue locally, and the fixed-box constraint forces the surrounding tissue to expand uniformly to conserve total area. Because the eigenstress is isotropic (pure pressure) and the inclusion is circular, the outside strain has no shear or angular dependence. This is exact within linear elasticity and does not require any far-field or multipole approximation.

Cooperative enhancement – When Region 2 is activated in this pre-strained tissue, each edge inside Region 2 starts from a uniform background strain

$$\varepsilon_{\text{bg}} = \frac{1}{2}\nabla\cdot\mathbf{u} = +\frac{p_0\,\phi}{2(B+G)}\,, \tag{S35}$$

independent of Region 2's location. The isotropy of the dilation field means that $\varepsilon_{mn} = \varepsilon_{\text{bg}}\,\delta_{mn}$, so all edges experience the same background strain regardless of their orientation.

This pre-strain modifies the ratcheting dynamics during Region 2's activation. To leading order, the cooperative enhancement is linear in ε_{bg}:

$$\Delta\Lambda_\infty = \eta\,\varepsilon_{\text{bg}} = \frac{\eta\,p_0\,\phi}{2(B+G)}\,, \tag{S36}$$

where $\eta = \partial\Lambda_{\text{final}}/\partial\varepsilon_{\text{bg}}$ is the mechanosensitive susceptibility, quantifying how efficiently a uniform background strain biases the tension accumulation in Region 2.

Substituting $p_0 = n_e\delta\Lambda_{\text{src}}\ell_0/2$ from (S26):

$$\Delta\Lambda_\infty = \frac{\eta\,n_e\,\delta\Lambda_{\text{src}}\,\ell_0}{4(B+G)}\,\frac{\pi R^2}{L^2}\,. \tag{S37}$$

In the discrete tissue, the eigenstress has a small deviatoric component from network disorder. This generates a distance-dependent contribution to $\Delta\Lambda(D)$. For a circular inclusion in a 2D isotropic elastic medium, the deviatoric strain outside the inclusion decays as $\sim R^2/r^2$. The full cooperative enhancement is therefore:

$$\Delta\Lambda(D) = A\left(\frac{R}{D}\right)^2 + \Delta\Lambda_\infty\,, \tag{S38}$$

where A is set by the deviatoric eigenstress amplitude and the susceptibility. Since the activated region contains many randomly oriented edges, the deviatoric component is suppressed by $\sim 1/\sqrt{N_{\text{edges}}}$ relative to the isotropic part, making $A \ll \Delta\Lambda_\infty$. This explains the weak distance dependence of $\Delta\Lambda(D)$.

S6 Structural features

Periodic bulk oscillations modify tissue structure through the adaptive coupling between junctional strain and tension. We quantify these structural changes by tracking the evolution of cell-scale geometric statistics after training. As shown in Fig. S4a, when $\tilde{k}_C > \tilde{k}_E$ cell areas develop a pronounced high-variance distribution, characterized by the simultaneous presence of cells with enlarged and shrunk areas. This increase in area heterogeneity results in the reduction in Poisson ratio reported in the main text. In contrast, when $\tilde{k}_C < \tilde{k}_E$, the area distribution narrows and concentrates around the mean area of 1, consistent with a more uniform confluent monolayer.

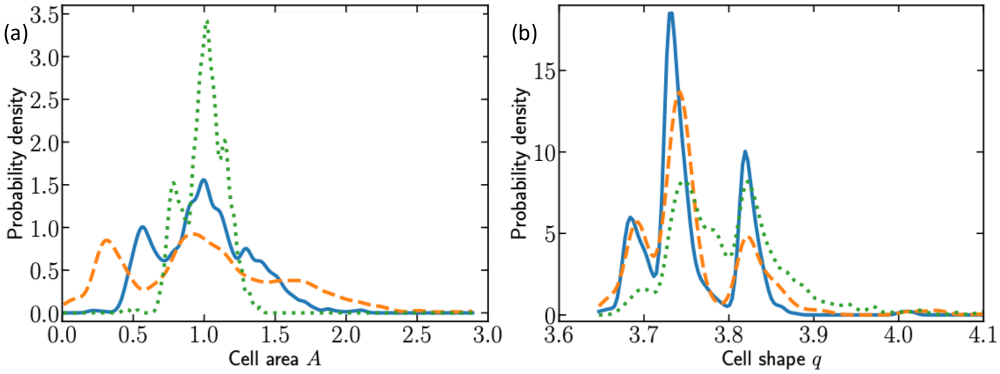

Fig. S4. (a) Probability density of cell areas. Shown are the initial distribution (solid blue), the post-training distribution for bulk-strain training with $\tilde{k}_C = 0.25$, $\tilde{k}_E = 0$ (dashed orange), and the post-training distribution for $\tilde{k}_C = 0$, $\tilde{k}_E = 0.25$ (dotted green). (b) Corresponding distributions of the cell shape index $q = P/\sqrt{A}$ computed from the same configurations as in panel (a). The data is averaged over 5 different random samples.

Figure S5 shows the evolution of cell-level pressure distributions under bulk-strain training. For $\tilde{k}_C > \tilde{k}_E$, the distribution develops pronounced positive and negative high-pressure tails, indicating the emergence of tiny and big cell populations as training progresses. In contrast, when $\tilde{k}_C < \tilde{k}_E$, cell pressures shift toward low but positive values, reflecting a more homogeneous cell area distribution. The resulting state is closer to a fluid-like, weakly compressed configuration, consistent with the increase in Poisson ratio reported in the main text.

Figure S6 shows the distributions of edge strain after bulk-strain training. The initial configuration has zero strain by construction. When $\tilde{k}_C > \tilde{k}_E$, the post-training distribution develops a pronounced heavy tail, indicating the presence of edges that undergo large relative elongation. In

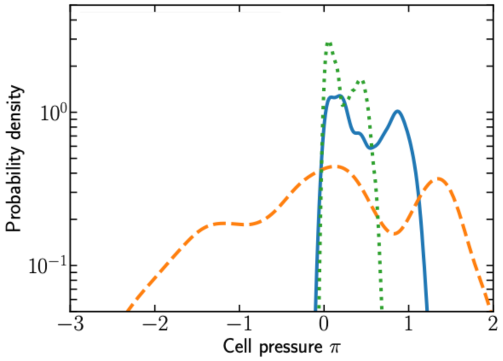

Fig. S5. Probability density of cell pressures $\pi_i = -2K_A(A_i - A_0)$ for the same configurations shown in Fig. S4. Displayed are the initial pressure distribution (solid blue), the post-training distribution for bulk-strain training with $\tilde{k}_C = 0.25$, $\tilde{k}_E = 0$ (dashed orange), and the post-training distribution for $\tilde{k}_C = 0$, $\tilde{k}_E = 0.25$ (dotted green). Data are averaged over 5 independent random samples.

contrast, for $\tilde{k}_C < \tilde{k}_E$, the strain distribution remains sharply peaked, reflecting a population of edges that experience only small deviations from their reference lengths.

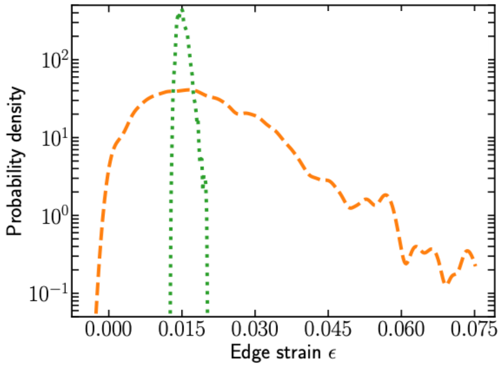

Fig. S6. Probability density of edge strains $\epsilon_{ij} = (\ell_{ij} - \ell^0_{ij})/\ell^0_{ij}$ for the trained tissues shown in Fig. S4. Displayed are the post-training strain distributions for bulk-strain training with $\tilde{k}_C = 0.25$, $\tilde{k}_E = 0$ (dashed orange) and for $\tilde{k}_C = 0$, $\tilde{k}_E = 0.25$ (dotted green). The initial configuration is not shown because all edges begin at zero strain. Data are averaged over 5 independent random samples.

S7 Effect of T1 transitions on trained mechanical response

In the main text, we show that cyclic bulk deformations can train the tissue Poisson ratio through junctional tension remodeling. Since cell–cell neighbor exchanges can alter the identity of individual junctions, we asked whether the trained mechanical response is sensitive to T1 transitions. This is particularly relevant because the memory in our model is stored in junctional tensions and rest lengths.

We tested this by training tissues under the same oscillatory bulk deformation protocol used in the main text, while varying the T1 cutoff length ℓ_{T1}. Increasing ℓ_{T1} causes edges to undergo T1 transitions at larger lengths and therefore increases the number of neighbor exchanges during training. This allows us to directly test whether enhanced topological rearrangements erase or significantly modify the trained Poisson ratio.

A subtle issue is how the tension of the newly created edge should be assigned after a T1 transition. Since this microscopic rule is not uniquely determined, we considered three post-T1 tension-assignment rules, shown schematically in Fig. S7. These rules have been proposed in prior work (2, 3). In case I, the newly created edge inherits the tension of the disappearing edge. In case II, the new edge is assigned the mean tension of the tissue, $\langle \Lambda \rangle$. In case III, the new edge tension is randomly resampled from the range of existing edge tensions in the tissue.

To increase the number of T1 events and make this a stringent test, we used a lower baseline contractility, $\Gamma_a = 0.05$, which makes the tissue softer and more susceptible to rearrangements. Figure S7b shows the cumulative number of T1 events, normalized by the number of cells, as a function of training time. As expected, increasing the T1 cutoff length increases the number of neighbor exchanges during training.

We then measured the normalized change in Poisson ratio, $\Delta\nu/\nu_0$, after training as a function of ℓ_{T1}. Results are shown for both remodeling regimes: $\tilde{k}_C > \tilde{k}_E$ and $\tilde{k}_E > \tilde{k}_C$ (Fig. S7c,d). In both regimes, the trained change in Poisson ratio depends only weakly on ℓ_{T1} over the range studied. Moreover, the three post-T1 tension-assignment rules produce nearly indistinguishable values of $\Delta\nu/\nu_0$.

These results indicate that the trained mechanical response is robust to occasional T1 transitions in the parameter regime considered here. Although individual neighbor exchanges can locally alter the microscopic tension pattern, they do not erase the tissue-scale mechanical response measured after relaxation. This robustness likely reflects two factors. First, the number of T1 events remains limited during the training protocol. Second, the reported Poisson ratio is computed after arresting the dynamics and relaxing the tissue to a force-balanced state, so the measured response reflects the collective remodeled tissue state rather than the detailed history of any single junction. Thus, while frequent topological rearrangements could in principle decorrelate junctional memory in a strongly fluidized tissue, the conclusions reported here are insensitive to reasonable choices of the post-T1 tension rule and to the range of T1 activity studied.

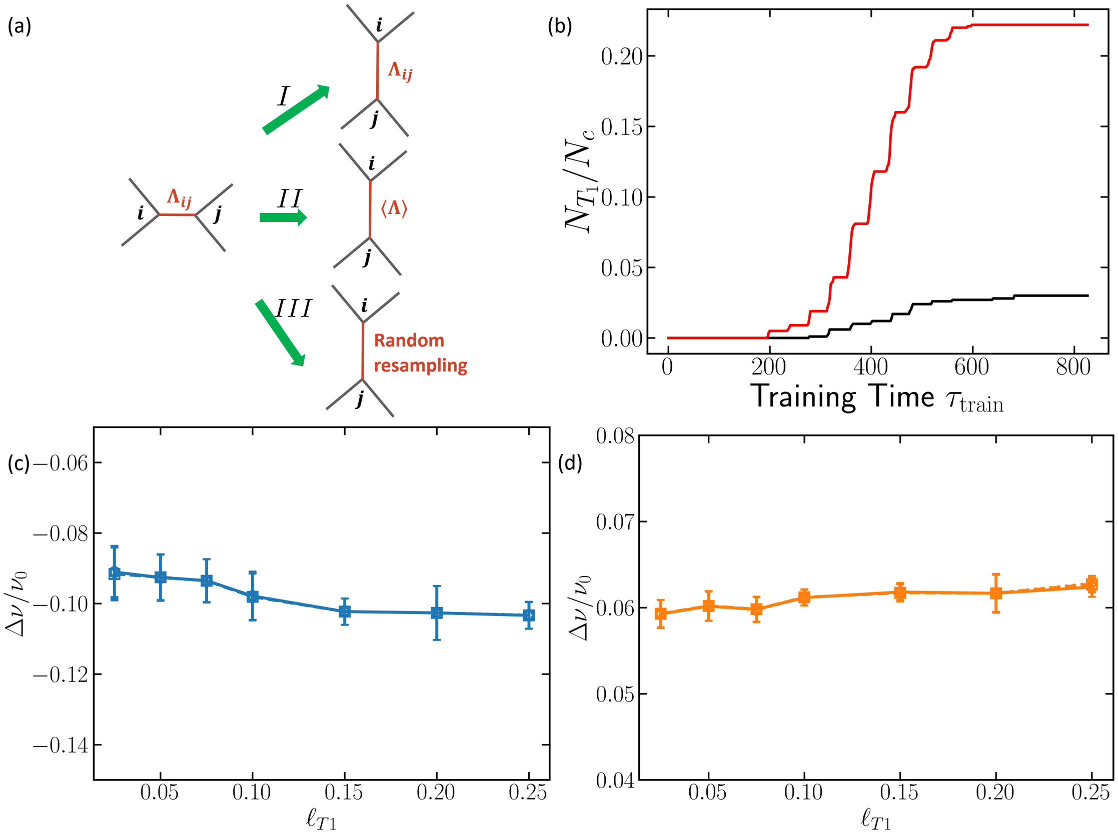

Fig. S7. Effect of T1 transitions on trained mechanical response. (a) Three post-T1 tension-assignment rules tested in the simulations: case I, the newly created edge inherits the tension of the disappearing edge; case II, the new edge is assigned the mean tension of the tissue, $\langle\Lambda\rangle$; and case III, the new edge tension is randomly resampled from the range of existing tissue tensions. (b) Cumulative number of T1 events, normalized by the number of cells, as a function of training time for case I. Increasing the T1 cutoff length increases the number of neighbor exchanges: black, $\ell_{T1} = 0.05$; red, $\ell_{T1} = 0.2$. (c,d) Normalized change in Poisson ratio, $\Delta\nu/\nu_0$, as a function of the T1 cutoff length ℓ_{T1} for (c) $\tilde{k}_C = 0.07$, $\tilde{k}_E = 0$, and (d) $\tilde{k}_C = 0$, $\tilde{k}_E = 0.07$. Circles, squares, and diamonds denote post-T1 rules I, II, and III, respectively. The symbols overlap closely, showing that the trained mechanical response is insensitive to the post-T1 tension-assignment rule over the range studied. Training was performed under oscillatory bulk deformation with strain amplitude $\varepsilon_B^{\mathrm{Max}} = 0.2$ and critical edge strain $\varepsilon_c = 0.01$. Tissues were trained for 20 oscillations with period $T = 40$, using $N = 200$ cells and baseline contractility $\Gamma_a = 0.05$.

S8 Minimal mean-field model for auxetic response

The bulk-training simulations in the main text show that when contraction-induced remodeling dominates over extension-induced remodeling, $\tilde{k}_C > \tilde{k}_E$, junctional tensions increase over training and the tissue Poisson ratio decreases, eventually becoming negative. To clarify the mechanical origin of this effect, we considered a single-cell mean-field vertex model with fixed, uniform edge tension.

We model the cell as a symmetric hexagon with horizontal half-width x and vertical span y. The six vertices are

$$(x,0), \quad (x/2, y/2), \quad (-x/2, y/2),$$
$$(-x,0), \quad (-x/2,-y/2), \quad (x/2,-y/2).$$

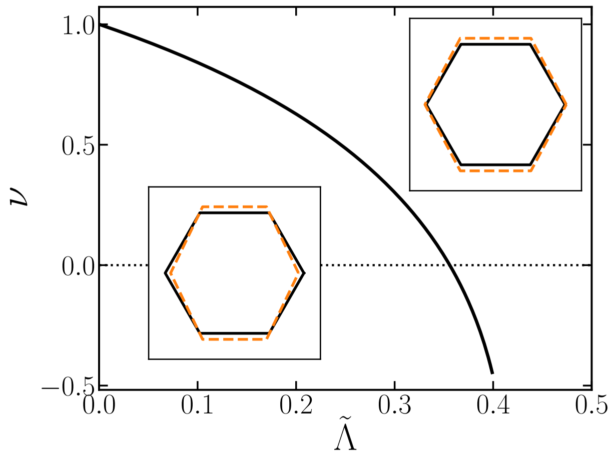

Fig. S8. Poisson ratio ν of the minimal hexagonal vertex model as a function of the dimensionless junctional tension $\tilde{\Lambda}$. Increasing $\tilde{\Lambda}$ lowers ν and eventually produces an auxetic response. Insets show representative relaxed deformations under a small vertical extension.

The cell energy is

$$E = K_A(A - A_0)^2 + \Lambda P, \tag{S39}$$

where A and P are the area and perimeter of the hexagon, K_A is the area modulus, A_0 is the preferred area, and Λ is a fixed, uniform edge tension. We use $K_A = 1$ and $A_0 = 1$, and define the dimensionless tension $\tilde{\Lambda} = \Lambda/(K_A A_0^{3/2})$.

For each value of $\tilde{\Lambda}$, we first found the isotropic relaxed state by minimizing Eq. (S39) over regular hexagons, with $y = \sqrt{3}x$. We then imposed a small extensional strain in the vertical direction, $y \to y(1+\varepsilon_y)$, and minimized the energy with respect to the horizontal degree of freedom x while keeping the imposed vertical strain fixed. The lateral strain was computed from the relaxed horizontal width, and the Poisson ratio was measured as $\nu = -\frac{\varepsilon_x}{\varepsilon_y}$. For the values reported in the main text, we used $\varepsilon_y = 0.01$ to remain in the linear-response regime. As $\tilde{\Lambda}$ increases, the Poisson ratio of the hexagonal cell decreases and eventually becomes negative (Fig. S8), showing that auxeticity can arise directly from the competition between area elasticity and junctional tension.

S9 Comparison between trained tissues and static high-tension vertex models

The minimal mean-field model above shows that sufficiently large fixed junctional tension can lower the Poisson ratio and eventually produce an auxetic response. This raises an important question: is the trained tissue generated by the remodeling dynamics physically equivalent to a static vertex model with high edge tensions? To address this point, we directly compared a trained adaptive tissue to a static fixed-tension control with a similar distribution of active edge tensions.

We first trained a tissue under cyclic bulk deformation in the contraction-dominated regime, $\tilde{k}_C > \tilde{k}_E$, where the Poisson ratio decreases and becomes negative. We then measured the distribution of active edge tensions Λ_{ij} in the final trained state. To construct a static control, we used the same vertex-model energy but held all edge tensions fixed and chose the fixed-tension values so that their distribution was similar to that of the trained tissue. Thus, both tissues are tension-dominated and both exhibit an auxetic response. The comparison below asks whether they nevertheless represent the same material state.

Figure S9a shows that the active edge-tension distributions of the trained tissue and the static control are similar by construction. The polygon distributions are also broadly similar, although the trained tissue shows a modest increase in highly coordinated cells and a small number of low-coordination cells (Fig. S9b). However, the cell-area distributions are more distinct. The trained tissue develops a broader area distribution, with enhanced populations of both small and large cells, whereas the static control has a narrower and more continuous distribution of cell areas (Fig. S9c).

A clearer distinction appears when the mean active tension around each cell is plotted against that cell's area. For each cell α, we define

$$\langle \Lambda \rangle_\alpha = \frac{1}{n_\alpha} \sum_{\langle ij \rangle \in \alpha} \Lambda_{ij}, \tag{S40}$$

where n_α is the number of edges of cell α. In both tissues, $\langle \Lambda \rangle_\alpha$ and A_α are anticorrelated, consistent with the fact that high junctional tensions tend to constrict cells. However, the trained tissue shows a much stronger organization of tension with respect to cell geometry. In the static control, the scatter of $\langle \Lambda \rangle_\alpha$ versus A_α is relatively continuous. In contrast, the trained tissue shows a more clustered structure, with distinct populations of small high-tension cells, large cells, and cells near the preferred area (Fig. S9d). Thus, even when the distribution of edge tensions is similar, the trained tissue places those tensions differently in the tissue. The remodeling rule therefore does not simply increase the amount of tension; it couples tension to the evolving cell geometry.

We next compared the rheological response of the two tissues. In a step-strain stress-relaxation test, we applied a small simple shear strain and then held the box shape fixed while allowing the vertices to relax. In the static fixed-tension control, the tissue relaxes through the fast overdamped vertex dynamics and then reaches a plateau, as expected for a passive elastic vertex model. In the trained tissue, however, the edge tensions and rest lengths continue to evolve, producing an additional slow relaxation contribution (Fig. S9e).

Finally, we subjected the two tissues to an oscillatory shear deformation, $\gamma(t) = \gamma_{\max} \sin(\omega t)$. In the static control, the response is reversible, without hysteresis. In contrast, when the trained tissue is sheared while the remodeling variables continue to evolve, the stress–strain curve exhibits a finite hysteresis loop (Fig. S9f). This indicates that the trained tissue dissipates mechanical work through its internal adaptive variables, whereas the static high-tension control behaves as a passive elastic material.

Together, these results show that the trained tissue is not physically equivalent to a static high-tension vertex model. Although both systems can be tension-dominated and auxetic, the trained tissue differs in the spatial organization of tension and in its dynamical rheological response.

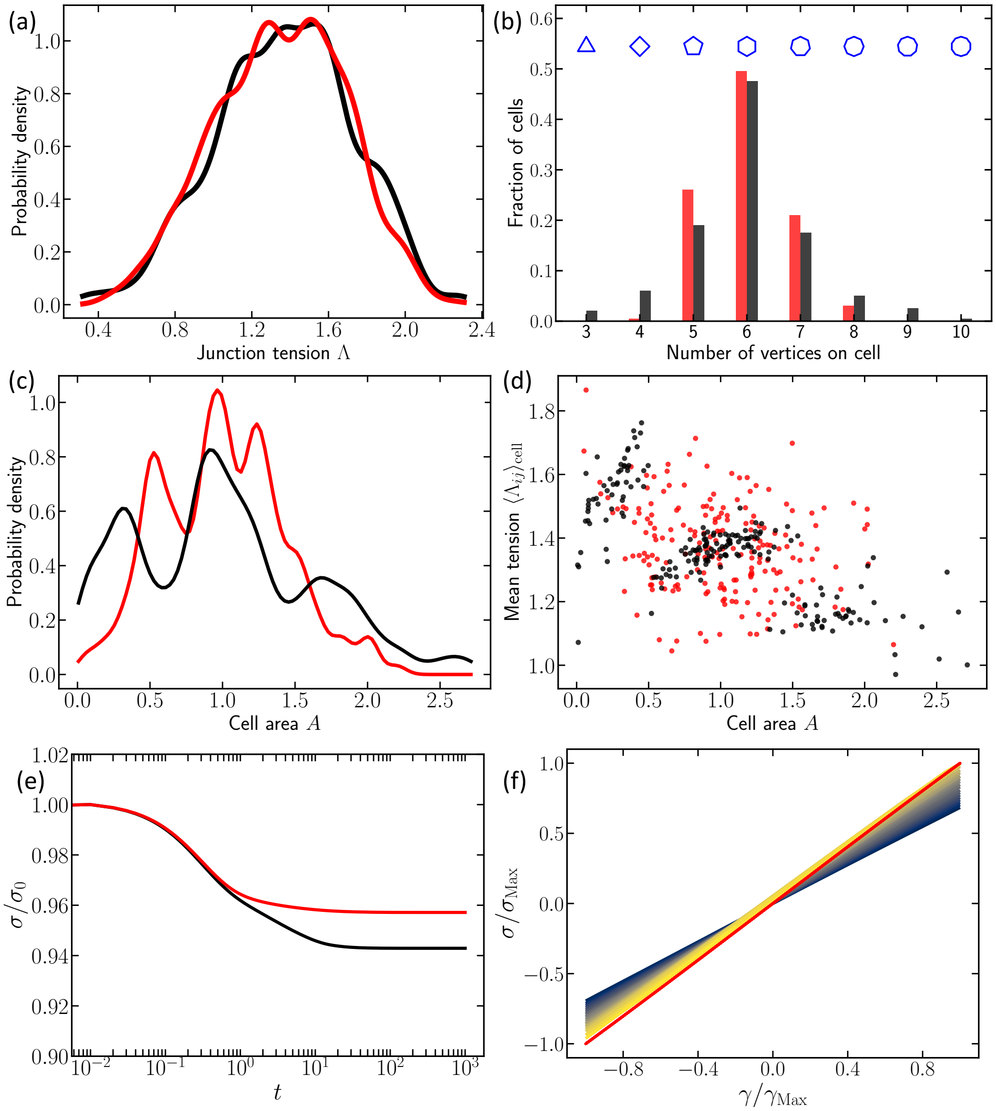

Fig. S9. Comparison between a trained adaptive tissue and a static fixed-tension vertex-model control. The trained tissue was generated by cyclic bulk deformation in the contraction-dominated regime, $\tilde{k}_C > \tilde{k}_E$, where training produces an auxetic response. The static control uses the same vertex-model energy but with fixed edge tensions and no tension or rest-length remodeling. (a) Active edge-tension distributions for the trained tissue (black) and the static control (red). The static control was chosen to have a similar marginal distribution of active edge tensions Λ_{ij} as the trained tissue. (b) Fraction of cells with a given number of vertices in the trained and static tissues. (c) Cell-area distributions. The trained tissue develops a broader area distribution, with enhanced populations of both small and large cells. (d) Mean active edge tension around each cell, $\langle \Lambda \rangle_\alpha$, as a function of cell area A_α. Although both systems show an anticorrelation between area and mean cell tension, the trained tissue displays a more organized, clustered structure, indicating that the remodeling rule couples junctional tension to cell geometry. (e) Stress relaxation after an imposed simple shear strain of magnitude $\gamma = 0.05$. The static control relaxes through the fast overdamped vertex dynamics and then reaches a plateau, whereas the trained tissue shows additional slow relaxation due to internal remodeling of edge tensions and rest lengths. (f) Oscillatory shear response under $\gamma(t) = \gamma_{\max} \sin(\omega t)$, with $\gamma_{\max} = 0.5$ and $\omega = 0.02$. The static control shows a reversible elastic response, while the trained tissue shows a finite hysteresis loop. Colors indicate progression through the trained-tissue cycle, from darker to lighter points.

S10 Non-affine character of the trained auxetic response

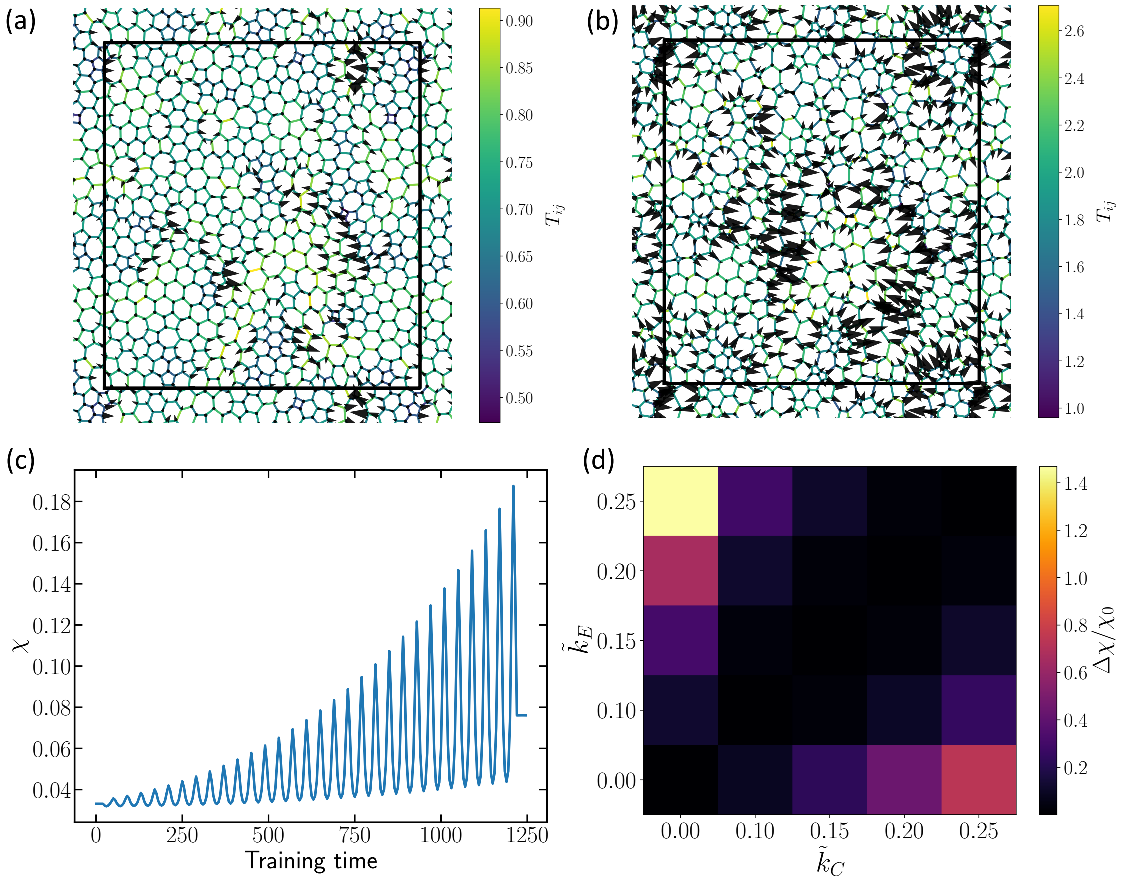

Fig. S10. (a) Non-affine displacement field during the Poisson-ratio measurement for the initial tissue configuration. Cell–cell junctions are colored by total tension T_{ij}, and black arrows show the non-affine component of the vertex displacement field. The arrows are rescaled for visualization. (b) Non-affine displacement field during the Poisson-ratio measurement for a trained tissue in the contraction-dominated regime, $\tilde{k}_C > \tilde{k}_E$. The arrows are rescaled for visualization by the same amount as in (a). (c) Time evolution of the non-affinity parameter χ during cyclic bulk training for a representative contraction-dominated simulation. (d) Normalized change in non-affinity, $\Delta\chi/\chi_0$, as a function of the contraction and extension remodeling rates, $\tilde{k}_C$ and $\tilde{k}_E$. Non-affinity increases most strongly when the remodeling rates are imbalanced, reflecting either heterogeneous structure in the contraction-dominated regime or a softer tissue in the extension-dominated regime.

To determine how the auxetic response manifests structurally in a disordered network, we analyzed the vertex displacement fields during the Poisson-ratio measurement. After imposing a small extensional strain ε_y and allowing relaxation, we computed the dimensionless non-affinity parameter (4)

$$\chi = \frac{\sum_{i=1}^{N_v} \left| \vec{u}_i - \vec{u}_i^{\text{af}} \right|^2}{N_v A_{\text{avg}} \varepsilon_y^2}, \tag{S41}$$

where N_v is the total number of vertices, A_{avg} is the mean cell area, and ε_y is the small imposed extensional strain. The vector $\vec{u}_i$ is the displacement of vertex i after mechanical relaxation, while $\vec{u}_i^{\text{af}}$ is the displacement that the same vertex would have under the corresponding homogeneous

affine deformation.

As the tissue trains into the highly tensed auxetic regime ($\tilde{k}_C > \tilde{k}_E$), the corresponding displacement field $\vec{u}_i - \vec{u}_i^{\mathrm{af}}$ becomes highly heterogeneous (Fig. S10a,b) and the non-affinity parameter χ continuously increases (Fig. S10c). Non-affine motion is concentrated near interfaces between small and larger neighboring cells, indicating that the auxetic response arises through the collective relaxation of a heterogeneous trained tissue rather than through a uniform affine expansion.

The non-affinity parameter generally increases during training when there is an imbalance between $\tilde{k}_C$ and $\tilde{k}_E$ (Fig. S10d). In the contraction-dominated regime, $\tilde{k}_C > \tilde{k}_E$, this increase reflects the development of strong structural heterogeneity; in the extension-dominated regime, $\tilde{k}_E > \tilde{k}_C$, it reflects a softer tissue. When $\tilde{k}_C$ and $\tilde{k}_E$ are comparable, the change in χ is much weaker. Thus, trained auxeticity is not associated with the suppression of non-affine motion, but rather with an organized non-affine response generated by tension remodeling and cell-scale heterogeneity.

S11 Effect of tension relaxation

The tension-remodeling framework described in the main text generates clear signatures of mechanical memory and adaptive response. In biological tissues, however, junctional tension relaxes over a finite timescale due to actomyosin turnover and adhesion dynamics. Rapid relaxation should diminish the influence of prior loading, whereas slow relaxation preserves it. All results presented so far correspond to the limit of no relaxation, $\tau_\Lambda = \infty$.

To examine how finite relaxation alters the dynamics, we vary τ_Λ in the tension dynamics, Eq. (S1), repeated here:

$$\frac{d\Lambda_{ij}}{dt} = -\,k(\varepsilon_{ij})(\ell_{ij} - \ell_{ij}^0) - \frac{1}{\tau_\Lambda}(\Lambda_{ij} - \Lambda_0), \tag{S42}$$

where the first term is the strain-dependent remodeling introduced in the main text, and the second term drives Λ_{ij} toward a baseline value Λ_0 over a timescale τ_Λ.

To examine how tension relaxation shapes mechanical memory, we first measured how a localized contractile pulse alters the shear modulus. Figure S11a shows the resulting change in shear modulus as a function of the inverse relaxation timescale τ_Λ^{-1}. When relaxation is fast, the imposed tension rapidly returns to its baseline value and the tissue retains no memory of the pulse, leading to essentially no change in G. As relaxation slows (smaller τ_Λ^{-1}), the effects of the contractile event persist, resulting in a finite increase in the shear modulus. In the limit of very slow relaxation, $\tau_\Lambda^{-1} \to 0$, ΔG approaches the no-relaxation behavior described in the main text.

We next quantified how finite tension relaxation modifies the cooperative, long-range memory effect described in the main text. In the absence of relaxation, tissues that previously experienced a contractile pulse in one region respond more strongly to a subsequent pulse applied in a distant region, reflecting persistent changes in the underlying tension field. To measure this cooperative effect, we computed the difference between the final average tension in the second activated region when a prior contractile event occurred elsewhere and the corresponding tension when no prior activation was applied. Figure S11b shows the normalized response $\Delta\Lambda/\Lambda_0$ in the second region as a function of the inverse relaxation timescale τ_Λ^{-1}. When relaxation is fast (large τ_Λ^{-1}), the cooperative effect vanishes: tension rapidly returns to its baseline value, eliminating any imprint of earlier contractile signals. As relaxation slows (smaller τ_Λ^{-1}), the cooperative response increases, indicating the accumulation of a finite mechanical memory. In the limit of very slow relaxation, the effect saturates to a plateau corresponding to the no-relaxation regime.

Figure S11c shows how the Poisson ratio changes in tissues trained under the bulk oscillation protocol described in the main text, plotted as a function of the inverse tension-relaxation timescale

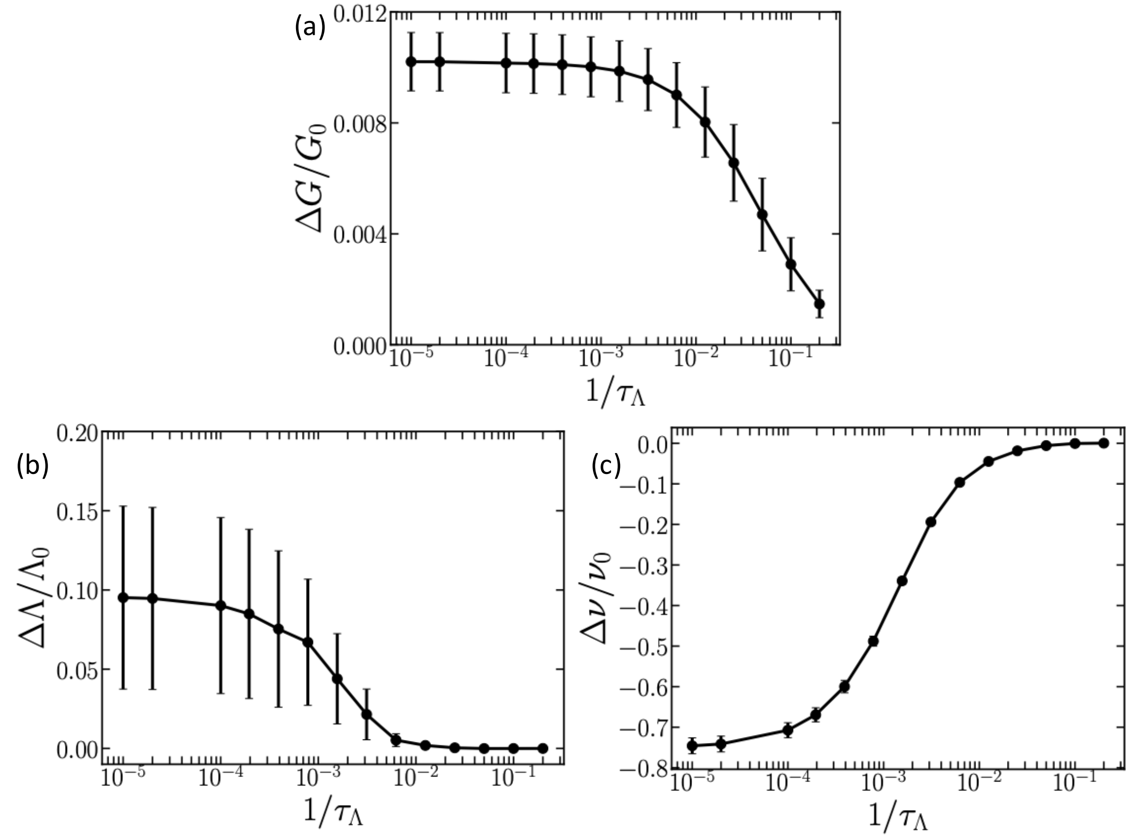

Fig. S11. (a) Normalized change in shear modulus $\Delta G/G_0$ as a function of the inverse tension-relaxation timescale τ_Λ^{-1}. The tissue is trained by applying a single contractile pulse in a central circular region of radius $R = L/6$ (with $L = \sqrt{N}$ and $N = 200$ cells). The active signal is increased from $\Gamma_a = 0.5$ to $\Gamma_a = 2.0$ for a duration $\mathcal{T} = 40$. Results are shown for contraction and extension remodeling rates $k_C = 0.2$ and $k_E = 0.05$. (b) Cooperative tension-memory response $\Delta\Lambda/\Lambda_0$ in a distant region as a function of τ_Λ^{-1}. The contractile signal is again pulsed between $\Gamma_a = 0.5$ and $\Gamma_a = 2.0$ for a duration $\mathcal{T} = 40$, but here applied repeatedly for 10 on–off cycles. The center-to-center separation between the previously activated region and the probe region is $D/L = 0.53$. The same remodeling parameters $k_C = 0.2$ and $k_E = 0.05$ are used. (c) Normalized change in Poisson ratio as a function of the inverse tension-relaxation timescale τ_Λ^{-1}, for tissues trained under the bulk oscillatory protocol described in the main text. Results are shown for contraction and extension remodeling rates $\tilde{k}_C = 0.2$ and $\tilde{k}_E = 0.05$. The bulk strain amplitude for this training is $\varepsilon_B = 0.2$ and the edge-level critical strain is $\varepsilon_c = 0.01$.

τ_Λ^{-1}. When tension relaxes rapidly (large τ_Λ^{-1}), the system effectively erases the history of loading, and the final mechanical state remains unchanged. In contrast, slow relaxation allows tension updates to accumulate, producing a finite shift in tissue properties and a persistent trained state. In biological epithelia, tension relaxation is governed by actin turnover and myosin motor dynamics, which operate on finite timescales. Memory over repeated deformation cycles therefore requires that remodeling outpace this relaxation, or that a slower process latch the remodeled state, as discussed in the main text.

S12 Effect of stochastic junctional tension fluctuations

To test whether stochastic fluctuations erase the trained mechanical response, we added noise directly to the edge-tension dynamics. Since mechanical memory in our model is stored in the remodeled junctional tensions and rest lengths, we modeled fluctuations in the active junctional tension rather than adding thermal noise to vertex positions. This choice follows the tension-fluctuation framework introduced in Ref. (5), where stochastic junctional tension fluctuations provide a mechanism for mechanical memory dissipation.

We decomposed the active junctional tension into a deterministic remodeling contribution and a fluctuating component,

$$\Lambda_{ij}^{\mathrm{tot}}(t) = \Lambda_{ij}(t) + \delta\Lambda_{ij}(t), \tag{S43}$$

where $\Lambda_{ij}(t)$ follows the strain-dependent remodeling dynamics described in the main text, while $\delta\Lambda_{ij}(t)$ evolves as an Ornstein–Uhlenbeck process,

$$\frac{d\delta\Lambda_{ij}}{dt} = -\frac{1}{\tau_{\mathrm{fluc}}}\delta\Lambda_{ij} + \sqrt{\frac{2\sigma_{\mathrm{fluc}}^2}{\tau_{\mathrm{fluc}}}}\,\xi_{ij}(t). \tag{S44}$$

Here σ_{fluc} is the fluctuation amplitude, τ_{fluc} is the persistence time of tension fluctuations, and $\xi_{ij}(t)$ is a Gaussian white-noise process. We use the notation $\Lambda_{ij}^{\mathrm{tot}}$ rather than T_{ij} to avoid confusion with the total edge tension used in the main text, where T_{ij} also includes the contractility contribution.

We repeated the cyclic bulk-training protocol and measured the normalized change in Poisson ratio, $\Delta\nu/\nu_0$, as a function of σ_{fluc}. Figure S12 shows that the trained response is not erased by the range of tension fluctuations studied. For small fluctuation amplitudes, the response is essentially unchanged. At larger fluctuation amplitudes, the magnitude of the trained response is modestly enhanced: in the contraction-dominated regime, $\tilde{k}_C > \tilde{k}_E$, the Poisson ratio decreases further, whereas in the extension-dominated regime, $\tilde{k}_E > \tilde{k}_C$, the Poisson ratio increases further.

This behavior is consistent with the thresholded nature of the remodeling rule. Tension fluctuations perturb individual edges across the strain thresholds more often during cyclic driving. As a result, they can increase the number of remodeling events without changing the direction selected by the deterministic rule. Thus, over the range studied here, stochastic junctional tension fluctuations do not erase the trained mechanical response.

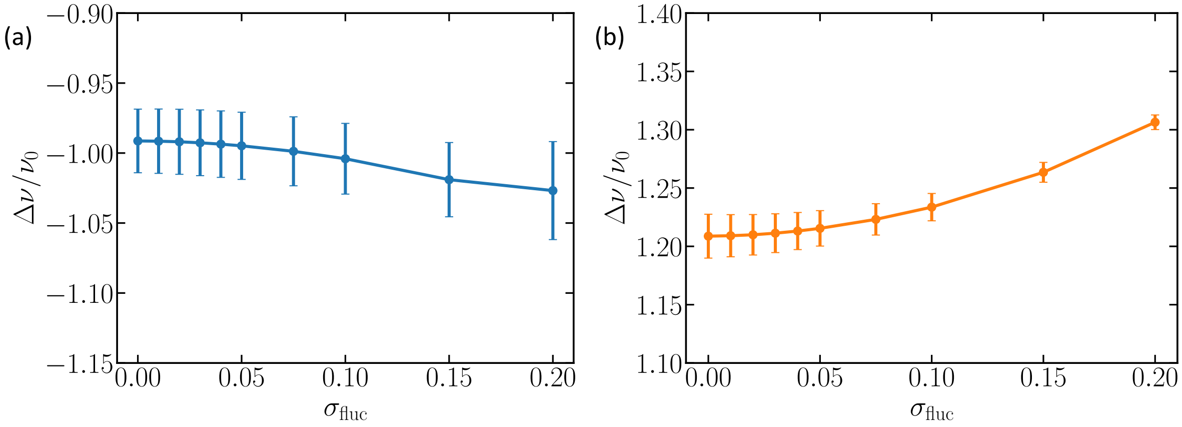

Fig. S12. Effect of junctional tension fluctuations on oscillatory bulk training. (a) Normalized change in Poisson ratio, $\Delta\nu/\nu_0$, as a function of the tension-fluctuation amplitude $\sigma_{\rm fluc}$ for the contraction-dominated regime, $\tilde{k}_C = 0.2$ and $\tilde{k}_E = 0$. (b) Corresponding result for the extension-dominated regime, $\tilde{k}_C = 0$ and $\tilde{k}_E = 0.2$. Simulations were performed using the cyclic bulk-training protocol with contractility $\Gamma_a = 1.0$, critical edge strain $\varepsilon_c = 0.01$, maximum bulk strain amplitude $\varepsilon_B^{\rm Max} = 0.2$, and 30 sinusoidal bulk-strain cycles with period $T = 40$. The tension-fluctuation persistence time is $\tau_{\rm fluc} = 0.4$, and the deterministic tension-relaxation time is taken to be infinite, $\tau_\Lambda = \infty$.

S13 Supplementary Movies

Movie S1. Global effects of local contractions. Time-lapse of a tissue subjected to pulsatile active contractility ($\Gamma_a(t)$) in a circular region of radius R at the tissue center, in the regime $k_C > k_E$. Cell–cell junctions are colored by their total tension T_{ij}. The shear modulus G, computed under this activation, increases over time, illustrating the stiffening behavior described in the main text.

Movie S2. Cooperative effects of local contractions. Time-lapse of a tissue subjected to pulsatile active contractility ($\Gamma_a(t)$) in two circular regions of radius R separated by a distance D, in the regime $k_C > k_E$. Cell–cell junctions are colored by their active tensions Λ_{ij}. The movie also shows the time evolution of the average active tension in each region, highlighting the enhanced response in the second region due to prior activation of the first, as quantified in the cooperative enhancement analysis in the main text.

Movie S3. Training tissues under oscillatory bulk deformation. Time-lapse of a tissue subjected to a sinusoidal bulk deformation signal $\varepsilon_B(t)$ in the regime $k_C > k_E$. Cell–cell junctions are colored by their tension T_{ij}. During training, junctional tensions grow and the tissue develops a heterogeneous structure with coexisting very large and very small cells. The Poisson ratio ν decreases over successive cycles and becomes negative, demonstrating the emergence of auxetic behavior discussed in the main text.

References

1. Kate E. Cavanaugh, Michael F. Staddon, Edwin Munro, Shiladitya Banerjee, and Margaret L. Gardel. RhoA Mediates Epithelial Cell Shape Changes via Mechanosensitive Endocytosis. *Developmental Cell*, 52(2):152–166.e5, January 2020. ISSN 1534-5807. doi: 10.1016/j.devcel.2019.12.002. URL `https://www.cell.com/developmental-cell/abstract/S1534-5807(19)31023-8`. Publisher: Elsevier.

2. Matej Krajnc. Solid–fluid transition and cell sorting in epithelia with junctional tension fluctuations. *Soft Matter*, 16(13):3209–3215, April 2020. ISSN 1744-6848. doi: 10.1039/C9SM02310K. URL `https://pubs.rsc.org/en/content/articlelanding/2020/sm/c9sm02310k`. Publisher: The Royal Society of Chemistry.

3. Takaki Yamamoto, Daniel M. Sussman, Tatsuo Shibata, and M. Lisa Manning. Non-monotonic fluidization generated by fluctuating edge tensions in confluent tissues. *Soft Matter*, 18(11):2168–2175, March 2022. ISSN 1744-6848. doi: 10.1039/D0SM01559H. URL `https://pubs-rsc-org.libezproxy2.syr.edu/en/content/articlelanding/2022/sm/d0sm01559h`. Publisher: The Royal Society of Chemistry.

4. Sadjad Arzash, Jordan L. Shivers, and Fred C. MacKintosh. Finite size effects in critical fiber networks. *Soft Matter*, 16(29):6784–6793, 2020. doi: 10.1039/D0SM00764A. URL `https://pubs.rsc.org/en/content/articlelanding/2020/sm/d0sm00764a`.

5. Fernanda Pérez-Verdugo and Shiladitya Banerjee. Tension remodeling regulates topological transitions in epithelial tissues. *PRX Life*, 1(2):023006, 2023.
